# Supplementary material for: Structural Homology-Based Drug Repurposing Approach for Targeting NSP12 SARS-CoV-2
Source: Molecules. 2022 Nov 10;27(22):7732. doi: 10.3390/molecules27227732 (PMC9694939; doi:10.3390/molecules27227732)
Supplement: Supplementary file 1 [file molecules-27-07732-s001.zip › molecules-1994099-supplementary.pdf]

Table S1. NSP-12 homologous structures predicted by DALI server.

Rank	PDB	Chain	Z_score	rmsd	lali	nres	% id	Description
1	6nus	A	64.5	0	715	715	100	NSP12
2	6nur	A	50.7	0.7	715	793	100	NSP12
3	3uqs	C	25.4	2.9	363	475	11	RNA-DEPENDENT RNA POLYMERASE
4	3ur0	B	25.1	2.9	366	476	11	RNA-DEPENDENT RNA POLYMERASE
5	4nrt	A	24.9	3.1	369	500	10	HNV-RDRP
6	3sfg	B	24.9	3	364	474	11	RNA POLYMERASE
7	3bso	A	24.9	3.3	369	479	10	RNA DEPENDENT RNA POLYMERASE
8	3upf	A	24.7	2.9	365	476	11	RNA-DEPENDENT RNA POLYMERASE
9	5y3d	F	24.7	2.9	364	481	11	RNA-DEPENDENT RNA POLYMERASE
10	3nai	B	24.6	2.9	365	486	11	RNA DEPENDENT RNA POLYMERASE
11	3uqs	B	24.6	2.9	366	482	11	RNA-DEPENDENT RNA POLYMERASE
12	1sh2	A	24.6	3.1	371	502	10	RNA POLYMERASE
13	3uqs	A	24.5	2.9	366	478	11	RNA-DEPENDENT RNA POLYMERASE
14	4o4r	A	24.4	2.9	363	476	12	RNA-DEPENDENT-RNA-POLYMERASE
15	3qid	C	24.4	2.9	366	492	11	RNA DEPENDENT RNA POLYMERASE
16	3ur0	C	24.4	3	362	474	11	RNA-DEPENDENT RNA POLYMERASE
17	3sfu	C	24.3	2.9	363	478	11	RNA POLYMERASE
18	3sfg	A	24.3	3	362	480	11	RNA POLYMERASE
19	2cjq	A	24.2	3.7	392	546	11	RNA-DIRECTED RNA POLYMERASE
20	4nru	A	24	2.9	363	477	11	RNA DEPENDENT RNA POLYMERASE
21	4lq9	A	23.9	3	368	501	10	RNA-DEPENDENT RNA-POLYMERASE
22	1wne	A	23.9	2.9	366	476	11	5'-R(*CP*AP*UP*GP*GP*GP*CP*C)-3'
23	2b43	D	23.8	3	367	501	10	NON-STRUCTURAL POLYPROTEIN
24	2wk4	B	23.6	3.2	376	502	12	PROTEASE-POLYMERASE P70
25	1sh0	A	23.6	3	372	502	10	RNA POLYMERASE
26	5jxs	A	23.5	2.9	367	476	11	RNA DEPENDENT RNA POLYMERASE
27	6gvv	A	23.4	2.8	365	480	11	RNA POLYMERASE 3D
28	4nru	C	23.3	2.9	366	478	11	RNA DEPENDENT RNA POLYMERASE
29	3nai	A	23.3	2.9	365	486	12	RNA DEPENDENT RNA POLYMERASE
30	3qid	B	23.2	2.9	365	492	11	RNA DEPENDENT RNA POLYMERASE
31	5y3d	C	23.2	2.8	364	480	11	RNA-DEPENDENT RNA POLYMERASE
32	5y3d	B	23.2	2.8	361	479	11	RNA-DEPENDENT RNA POLYMERASE
33	2d7s	A	23.2	2.8	365	474	11	RNA-DEPENDENT RNA POLYMERASE
34	4nru	E	23.1	2.9	364	477	11	RNA DEPENDENT RNA POLYMERASE
35	4nz0	F	23.1	2.8	361	460	12	GENOME POLYPROTEIN
36	4wfy	A	23.1	2.7	351	462	14	RNA-DIRECTED RNA POLYMERASE
37	3sfu	B	23	2.9	368	487	11	RNA POLYMERASE
38	4nru	F	23	2.9	361	473	11	RNA DEPENDENT RNA POLYMERASE
39	4nz0	E	23	2.8	359	460	13	GENOME POLYPROTEIN

40	3nah	A	23	3	368	486	11	RNA DEPENDENT RNA POLYMERASE
41	3nah	C	22.9	2.9	364	485	11	RNA DEPENDENT RNA POLYMERASE
42	3qid	A	22.9	3	364	492	11	RNA DEPENDENT RNA POLYMERASE
43	3ur0	A	22.9	2.9	362	475	11	RNA-DEPENDENT RNA POLYMERASE
44	4wfx	A	22.9	2.7	348	462	15	RNA-DIRECTED RNA POLYMERASE
45	3klv	A	22.9	2.8	364	476	11	3D POLYMERASE
46	4qpx	A	22.9	3.2	368	495	11	POLYPROTEIN
47	3upf	B	22.9	2.9	365	483	11	RNA-DEPENDENT RNA POLYMERASE
48	1khv	B	22.9	3.2	384	497	13	RNA-DIRECTED RNA POLYMERASE
49	2e9t	A	22.8	2.8	364	474	11	5'-R(P*UP*AP*GP*GP*GP*CP*CP*C)-3'
50	2uuw	A	22.8	3.2	377	490	11	RNA-DIRECTED RNA POLYMERASE
51	3ol6	I	22.8	2.9	355	461	15	POLYMERASE
52	3olb	A	22.8	3	356	461	15	POLYMERASE
53	3h5y	A	22.8	3.4	366	474	10	RNA DEPENDENT RNA POLYMERASE
54	4k4t	A	22.8	3	358	462	14	RNA-DIRECTED RNA POLYMERASE 3D-POL
55	3nah	B	22.8	2.9	368	485	11	RNA DEPENDENT RNA POLYMERASE
56	4k4t	E	22.7	3	358	462	14	RNA-DIRECTED RNA POLYMERASE 3D-POL
57	5yf7	A	22.7	3.3	425	650	13	RDRP CATALYTIC
58	1s4f	A	22.7	3.1	362	583	12	RNA-DEPENDENT RNA POLYMERASE
59	3h5x	A	22.7	3.3	366	470	10	RNA DEPENDENT RNA POLYMERASE
60	2im0	A	22.7	3	356	461	14	POLIOVIRUS POLYMERASE
61	6ae5	A	22.7	3.3	418	644	13	RDRP CATALYTIC
62	3ola	M	22.6	2.9	357	461	15	POLYMERASE
63	4nz0	C	22.6	2.8	358	460	13	GENOME POLYPROTEIN
64	4r0e	A	22.6	2.9	354	461	14	RNA-DIRECTED RNA POLYMERASE
65	4nlv	A	22.6	3	357	461	15	RNA-DIRECTED RNA POLYMERASE 3D-POL
66	5f8m	A	22.6	3	356	462	15	GENOME POLYPROTEIN
67	1sh3	B	22.5	3.1	369	503	10	RNA POLYMERASE
68	3bsn	A	22.5	3.4	367	479	10	RNA DEPENDENT RNA POLYMERASE
69	5y6z	A	22.5	3	355	457	15	GENOME POLYPROTEIN
70	2b43	B	22.4	3	367	501	10	NON-STRUCTURAL POLYPROTEIN
71	5y3d	E	22.4	2.8	364	483	11	RNA-DEPENDENT RNA POLYMERASE
72	6ae6	A	22.3	3.3	422	647	13	RDRP CATALYTIC
73	1s4f	B	22.2	3.1	364	583	12	RNA-DEPENDENT RNA POLYMERASE
74	2b43	C	22.2	3	369	501	10	NON-STRUCTURAL POLYPROTEIN
75	4o4r	C	22.1	3	364	474	11	RNA-DEPENDENT-RNA-POLYMERASE
76	3upf	C	22.1	2.9	362	471	12	RNA-DEPENDENT RNA POLYMERASE
77	1sh3	A	22.1	3	366	502	9	RNA POLYMERASE
78	5yf5	A	22	3.4	423	641	13	RDRP CATALYTIC

79	5y6r	A	21.9	3.4	424	667	12	GENOME POLYPROTEIN
80	1s4f	C	21.9	3.4	362	581	12	RNA-DEPENDENT RNA POLYMERASE
81	4wyw	A	21.8	2.8	364	479	11	RNA-DIRECTED RNA POLYMERASE 3D-POL
82	5yf8	A	21.7	3.4	399	645	12	RDRP CATALYTIC
83	1khv	A	21.6	3.2	384	493	13	RNA-DIRECTED RNA POLYMERASE
84	4zp8	A	21.6	2.8	349	454	14	RNA-DEPENDENT RNA POLYMERASE
85	5tsn	A	21.4	3.2	371	500	11	NORWALK VIRUS POLYMERASE
86	3ol9	I	21.3	3	357	461	15	POLYMERASE
87	4nlr	A	21.3	3	356	461	15	RNA-DIRECTED RNA POLYMERASE 3D-POL
88	3n6l	A	21.2	2.9	355	462	15	RNA-DEPENDENT RNA POLYMERASE
89	6ae6	B	21.1	3.7	425	647	12	RDRP CATALYTIC
90	3nl0	A	21	2.8	364	475	11	3D POLYMERASE
91	4wtj	A	21	3.8	365	555	12	RNA TEMPLATE AUCC
92	3nma	A	21	2.8	365	476	11	3D POLYMERASE
93	1yvz	A	21	3.8	361	548	11	RNA DEPENDENT RNA POLYMERASE
94	3kms	A	20.8	2.8	364	476	11	3D POLYMERASE
95	1tp7	C	20.8	2.8	349	460	14	GENOME POLYPROTEIN
96	5i62	A	20.7	3.1	339	507	12	POTENTIAL RNA-DEPENDENT RNA POLYMERASE
97	3koa	A	20.7	2.8	365	476	11	3D POLYMERASE
98	4k50	A	20.7	2.8	349	460	15	RNA POLYMERASE 3D-POL
99	4wzq	A	20.7	2.8	365	476	11	RNA DEPENDENT-RNA POLYMERASE 3D
100	6gvy	A	20.7	2.8	365	475	11	GENOME POLYPROTEIN
101	2ec0	A	20.7	2.8	364	470	11	5'-R(P*AP*UP*GP*GP*GP*CP*CP*C)-3'
102	5zit	A	20.7	2.9	355	456	14	RDRP
103	3nai	C	20.6	2.9	368	486	11	RNA DEPENDENT RNA POLYMERASE
104	5ccv	F	20.6	3.8	417	851	11	RNA-DIRECTED RNA POLYMERASE NS5
105	5f8n	A	20.6	3	354	462	15	GENOME POLYPROTEIN
106	4x2b	A	20.5	2.8	364	477	11	RNA DEPENDENT RNA POLYMERASE
107	1khw	B	20.5	3.2	384	497	13	RNA-DIRECTED RNA POLYMERASE
108	4o4r	B	20.5	2.9	363	480	12	RNA-DEPENDENT-RNA-POLYMERASE
109	4nly	A	20.5	3	355	461	14	RNA-DIRECTED RNA POLYMERASE 3D-POL
110	4k50	I	20.5	2.8	347	460	15	RNA POLYMERASE 3D-POL
111	5ccv	B	20.5	3.7	426	852	11	RNA-DIRECTED RNA POLYMERASE NS5
112	5n8x	A	20.5	2.8	364	475	11	3D POLYMERASE
113	4zpb	A	20.5	2.7	351	462	14	RNA-DIRECTED RNA POLYMERASE
114	4y3c	C	20.4	2.8	359	460	13	3D POLYMERASE
115	3vqs	B	20.4	4	366	557	12	RNA-DIRECTED RNA POLYMERASE
116	5hmw	A	20.4	3.5	399	591	11	RNA-DIRECTED RNA POLYMERASE NS5

117	4k6m	A	20.3	5.4	422	889	13	POLYPROTEIN
118	3vqs	D	20.3	4.1	369	553	13	RNA-DIRECTED RNA POLYMERASE
119	6mvo	A	20.3	4	351	562	15	RNA-DIRECTED RNA POLYMERASE
120	6i7p	E	20.3	13.1	435	884	12	NS5
121	3ol9	A	20.2	2.9	355	461	14	POLYMERASE
122	3qgd	A	20.2	4	350	538	13	RNA-DIRECTED RNA POLYMERASE
123	3qgf	A	20.2	4.1	354	534	13	RNA-DIRECTED RNA POLYMERASE
124	2fvc	A	20.2	3.8	350	563	13	POLYPROTEIN
125	4y2c	A	20.2	3	360	460	12	GENOME POLYPROTEIN
126	6izz	A	20.2	3.5	402	568	10	GENOME POLYPROTEIN
127	3gnw	B	20.1	4	364	568	12	RNA-DIRECTED RNA POLYMERASE
128	4aex	A	20.1	4.1	364	563	12	RNA-DIRECTED RNA POLYMERASE
129	3qge	A	20.1	4.2	353	541	13	RNA-DIRECTED RNA POLYMERASE
130	3h98	A	20.1	3.8	355	551	12	RNA-DIRECTED RNA POLYMERASE
131	4aep	A	20.1	4.1	364	563	12	RNA-DIRECTED RNA POLYMERASE
132	1yuy	A	20.1	4	364	558	12	RNA-DEPENDENT RNA POLYMERASE
133	3i5k	A	20.1	4	351	566	13	RNA-DIRECTED RNA POLYMERASE
134	1gx5	A	20.1	4	367	530	12	RNA-DIRECTED RNA POLYMERASE
135	4k4s	A	20.1	3	358	462	14	RNA-DIRECTED RNA POLYMERASE 3D-POL
136	3gnv	A	20	4	364	563	12	RNA-DIRECTED RNA POLYMERASE
137	3frz	A	20	4.1	364	562	12	RNA-DIRECTED RNA POLYMERASE
138	3u4o	A	20	4	363	563	12	RNA-DIRECTED RNA POLYMERASE
139	3ol6	E	20	3	356	461	15	POLYMERASE
140	3qgd	B	20	3.9	351	515	13	RNA-DIRECTED RNA POLYMERASE
141	1csj	B	20	3.9	363	531	12	HEPATITIS C VIRUS RNA POLYMERASE (NS5B)
142	1csj	A	20	3.9	364	531	12	HEPATITIS C VIRUS RNA POLYMERASE (NS5B)
143	3qgf	B	20	4.2	354	533	13	RNA-DIRECTED RNA POLYMERASE
144	2ax1	A	20	4	365	554	12	GENOME POLYPROTEIN
145	5trj	A	20	4.2	354	551	13	RNA-DEPENDENT RNA POLYMERASE
146	2ax1	B	20	4	365	561	13	GENOME POLYPROTEIN
147	4oow	A	19.9	4	365	555	12	RNA-DIRECTED RNA POLYMERASE
148	2zku	C	19.9	4	367	563	12	GENOME POLYPROTEIN
149	4nru	D	19.9	2.9	364	479	11	RNA DEPENDENT RNA POLYMERASE
150	1os5	A	19.9	3.9	361	563	12	HEPATITIS C VIRUS NS5B RNA POLYMERASE
151	3hhk	B	19.9	4	360	563	12	HCV NS5 POLYMERASE
152	3olb	E	19.9	3	356	461	15	POLYMERASE
153	3tyv	A	19.9	3.9	352	563	14	RNA-DIRECTED RNA POLYMERASE
154	1gx6	A	19.9	4	368	531	12	RNA-DIRECTED RNA POLYMERASE

155	2dxs	A	19.9	4	354	517	13	GENOME POLYPROTEIN
156	2o5d	B	19.9	3.9	363	561	12	HCV
157	3bsa	B	19.9	3.8	356	557	12	RNA-DIRECTED RNA POLYMERASE
158	2ax0	A	19.9	4	365	554	12	GENOME POLYPROTEIN
159	5y3d	D	19.9	2.9	364	479	11	RNA-DEPENDENT RNA POLYMERASE
160	1yvx	A	19.9	3.9	362	548	12	RNA DEPENDENT RNA POLYMERASE
161	4y3c	A	19.8	2.9	362	460	12	3D POLYMERASE
162	3qge	B	19.8	4.1	355	531	12	RNA-DIRECTED RNA POLYMERASE
163	2xi2	A	19.8	4	351	562	13	RNA-DIRECTED RNA POLYMERASE
164	2wrm	A	19.8	3.8	363	531	12	RNA-DIRECTED RNA POLYMERASE
165	4nlo	A	19.8	3	357	461	15	RNA-DIRECTED RNA POLYMERASE 3D-POL
166	4k4z	A	19.8	2.8	351	462	15	RNA-DEPENDENT RNA POLYMERASE
167	4eaw	B	19.8	4	366	559	12	RNA-DIRECTED RNA POLYMERASE
168	5hmz	A	19.8	3.5	400	585	11	RNA-DIRECTED RNA POLYMERASE NS5
169	2dxs	B	19.8	4.1	355	517	12	GENOME POLYPROTEIN
170	1khw	A	19.8	3.1	383	493	13	RNA-DIRECTED RNA POLYMERASE
171	5twn	A	19.8	3.9	342	550	14	NS5B RNA- DEPENDENT RNA POLYMERASE
172	4nru	B	19.7	2.9	365	482	11	RNA DEPENDENT RNA POLYMERASE
173	3skh	A	19.7	4	363	558	12	HCV NS5B RNA_DEPENDENT RNA POLYMERASE
174	3cde	A	19.7	3.9	361	557	12	RNA-DIRECTED RNA POLYMERASE
175	3u4o	B	19.7	3.9	359	558	12	RNA-DIRECTED RNA POLYMERASE
176	3hky	B	19.7	3.9	363	560	12	RNA-DIRECTED RNA POLYMERASE
177	3upi	B	19.7	3.9	349	558	13	RNA-DIRECTED RNA POLYMERASE
178	3i5k	D	19.7	4	351	566	13	RNA-DIRECTED RNA POLYMERASE
179	3q0z	B	19.7	3.9	342	545	14	RNA-DIRECTED RNA POLYMERASE
180	4wtd	A	19.7	3.6	366	534	12	RNA PRIMER TEMPLATE AUAAAUUU
181	4k50	E	19.7	2.8	346	460	15	RNA POLYMERASE 3D-POL
182	1nhv	B	19.7	3.9	363	561	12	
183	3cj4	A	19.7	4	364	558	12	RNA-DIRECTED RNA POLYMERASE
184	5tri	B	19.7	4.1	355	545	13	NS5B RNA-DEPENDENT RNA POLYMERASE
185	2i1r	B	19.7	3.9	361	561	12	RNA-DIRECTED RNA POLYMERASE (NS5B) (P68)
186	5y3d	A	19.7	2.9	364	479	11	RNA-DEPENDENT RNA POLYMERASE
187	5f8g	A	19.7	3.1	357	462	15	GENOME POLYPROTEIN
188	3i5k	B	19.6	4	364	566	12	RNA-DIRECTED RNA POLYMERASE
189	2xi2	C	19.6	4	363	561	13	RNA-DIRECTED RNA POLYMERASE
190	3u4r	B	19.6	3.9	359	558	12	RNA-DIRECTED RNA POLYMERASE
191	4iqx	A	19.6	2.8	364	476	11	3D POLYMERASE

192	2xi3	A	19.6	4.1	364	562	13	RNA-DIRECTED RNA POLYMERASE
193	4wtk	A	19.6	3.8	366	553	12	RNA TEMPLATE AGCC
194	3sfg	C	19.6	2.9	366	488	11	RNA POLYMERASE
195	3d5m	A	19.6	3.9	361	559	12	RNA-DIRECTED RNA POLYMERASE
196	4y3c	D	19.6	2.9	362	460	12	3D POLYMERASE
197	2xwy	A	19.6	4	355	507	13	RNA-DIRECTED RNA POLYMERASE
198	3i5k	C	19.6	4	351	566	13	RNA-DIRECTED RNA POLYMERASE
199	6h80	A	19.6	3.6	400	558	11	GENOME POLYPROTEIN
200	2hwi	B	19.6	4	363	561	12	RNA-DIRECTED RNA POLYMERASE (NS5B) (P68)
201	3cj0	A	19.6	3.8	355	562	12	RNA-DIRECTED RNA POLYMERASE
202	5uj2	A	19.6	3.8	364	534	13	RNA (5'-R(*AP*UP*AP*AP*AP*UP*UP*U)-3')
203	5trk	B	19.6	4.2	358	545	13	GENOME POLYPROTEIN
204	5pzl	B	19.6	4	344	545	14	RNA-DIRECTED RNA POLYMERASE
205	5czb	B	19.6	4	363	557	12	NS5B
206	3phe	D	19.5	3.9	364	558	12	HCV ENCODED NONSTRUCTURAL 5B PROTEIN
207	1u09	A	19.5	2.8	364	476	11	POLYPROTEIN
208	3kmq	A	19.5	2.8	365	476	11	3D POLYMERASE
209	4tn2	A	19.5	4.1	364	561	12	GENOME POLYPROTEIN
210	4mkb	A	19.5	3.9	362	557	12	RNA-DIRECTED RNA POLYMERASE
211	4nz0	A	19.5	2.9	363	460	12	GENOME POLYPROTEIN
212	3nky	A	19.5	2.8	365	476	11	3D POLYMERASE
213	3kna	A	19.5	2.8	365	476	11	3D POLYMERASE
214	3ska	B	19.5	4	362	558	12	HCV NS5B RNA_DEPENDENT RNA POLYMERASE
215	2hai	A	19.5	4	364	562	12	HEPATITIS C VIRUS NS5B RNA POLYMERASE
216	5tfr	B	19.5	12.5	433	883	10	GENOME POLYPROTEIN
217	2ec0	D	19.5	2.8	365	470	11	5'-R(P*AP*UP*GP*GP*GP*CP*CP*C)-3'
218	2wcx	A	19.4	3.9	356	512	13	RNA-DIRECTED RNA POLYMERASE
219	4kb7	B	19.4	4	362	548	12	HCV POLYMERASE
220	4tlr	A	19.4	4.1	364	564	12	NS5B
221	4nz0	D	19.4	2.8	360	460	11	GENOME POLYPROTEIN
222	4k4x	I	19.4	2.8	350	463	15	RNA-DEPENDENT RNA POLYMERASE
223	2xym	A	19.4	3.9	353	563	12	RNA-DIRECTED RNA POLYMERASE
224	2e9t	D	19.4	2.8	364	474	11	5'-R(P*UP*AP*GP*GP*GP*CP*CP*C)-3'
225	4dru	B	19.4	4.1	363	559	12	RNA-DIRECTED RNA POLYMERASE
226	4ke5	A	19.4	4	364	548	12	HCV POLYMERASE
227	3sfu	A	19.4	3	366	480	11	RNA POLYMERASE
228	4wzm	A	19.4	2.8	365	475	11	RNA DEPENDENT RNA POLYMERASE
229	2ax0	B	19.4	4	365	557	12	GENOME POLYPROTEIN

230	5xe0	A	19.4	2.9	354	457	14	GENOME POLYPROTEIN
231	6ae4	A	19.4	3.5	423	637	13	RDRP CATALYTIC
232	2jc0	A	19.4	3.9	362	557	12	RNA-DEPENDENT RNA-POLYMERASE
233	2jc0	B	19.4	3.9	360	555	12	RNA-DEPENDENT RNA-POLYMERASE
234	2hwh	A	19.4	3.9	363	559	12	RNA-DIRECTED RNA POLYMERASE (NS5B) (P68)
235	2f8e	X	19.4	2.8	364	474	11	RNA-DPENDENT RNA POLYMERASE
236	4zp6	A	19.4	2.7	347	454	14	GENOME POLYPROTEIN
237	5jjs	A	19.4	3.7	413	850	11	GENOME POLYPROTEIN
238	1s48	A	19.3	3.2	362	588	12	RNA-DEPENDENT RNA POLYMERASE
239	4mkb	B	19.3	3.9	363	557	12	RNA-DIRECTED RNA POLYMERASE
240	4mia	A	19.3	3.9	352	546	13	RNA-DIRECTED RNA POLYMERASE
241	4j02	A	19.3	3.9	363	559	12	GENOME POLYPROTEIN
242	3cvk	A	19.3	3.9	361	557	12	RNA-DIRECTED RNA POLYMERASE
243	4ry4	B	19.3	4	365	565	12	HCV J4 RNA POLYMERASE (NS5B)
244	1s49	A	19.3	3.2	362	588	12	RNA-DEPENDENT RNA POLYMERASE
245	4kai	B	19.3	4.1	365	551	12	HCV POLYMERASE
246	4ry4	A	19.3	3.9	364	566	12	HCV J4 RNA POLYMERASE (NS5B)
247	4ry5	B	19.3	3.9	353	565	13	HCV J4 RNA POLYMERASE (NS5B)
248	3ddk	A	19.3	2.7	352	462	14	RNA POLYMERASE B3 3DPOL
249	4k6m	B	19.3	10	433	889	13	POLYPROTEIN
250	3ol6	M	19.3	2.9	355	461	14	POLYMERASE
251	6mvk	B	19.3	4	362	550	12	HCV POLYMERASE
252	4zpa	A	19.3	2.7	351	462	15	RNA-DIRECTED RNA POLYMERASE
253	5n95	A	19.3	2.8	364	476	11	3D POLYMERASE
254	5u0c	E	19.3	3.4	413	622	12	NS5 RNA POLYMERASE DOMAIN
255	2hwh	B	19.3	4	364	561	12	RNA-DIRECTED RNA POLYMERASE (NS5B) (P68)
256	1yvf	A	19.3	4.1	367	564	12	HCV NS5B POLYMERASE
257	2gir	B	19.3	4	363	557	12	RNA-DIRECTED RNA POLYMERASE
258	3cj2	A	19.3	3.9	356	557	12	RNA-DIRECTED RNA POLYMERASE
259	1xr6	A	19.3	2.7	348	460	15	GENOME POLYPROTEIN
260	4zp7	A	19.3	2.6	348	456	15	GENOME POLYPROTEIN
261	5jjr	A	19.3	3.6	412	846	10	GENOME POLYPROTEIN
262	4zpd	A	19.3	2.8	355	462	14	RNA-DIRECTED RNA POLYMERASE
263	2zku	A	19.2	4	365	562	12	GENOME POLYPROTEIN
264	4wfz	A	19.2	2.6	350	462	15	RNA-DIRECTED RNA POLYMERASE
265	4wyl	A	19.2	2.8	364	478	11	RNA-DIRECTED RNA POLYMERASE 3D-POL
266	3hhk	A	19.2	4	363	563	12	HCV NS5 POLYMERASE
267	4khm	A	19.2	4	352	562	14	HCV POLYMERASE
268	4nz0	B	19.2	2.8	359	460	12	GENOME POLYPROTEIN

269	2who	B	19.2	3.9	363	531	12	RNA-DIRECTED RNA POLYMERASE
270	4khr	B	19.2	4.2	368	562	13	NS5B RNA-DEPENDENT RNA POLYMERASE
271	2xxd	A	19.2	4	353	563	12	RNA-DIRECTED RNA POLYMERASE
272	5wz3	A	19.2	3.6	395	565	12	NS5 RDRP
273	2xhw	A	19.2	4.1	365	560	12	RNA-DIRECTED RNA POLYMERASE
274	4y3c	E	19.2	2.8	362	460	12	3D POLYMERASE
275	4y3c	F	19.2	2.9	362	460	13	3D POLYMERASE
276	2e9r	X	19.2	2.8	366	475	11	5'-R(*CP*AP*UP*GP*GP*GP*CP*CP*C)-3'
277	5ziu	A	19.2	2.9	355	457	14	RDRP
278	1z4u	A	19.2	4	364	564	12	HCV NS5B POLYMERASE
279	3cj5	B	19.2	4.1	362	563	12	RNA-DIRECTED RNA POLYMERASE
280	2gc8	B	19.2	4	362	555	12	RNA-DIRECTED RNA POLYMERASE
281	2qe5	B	19.2	3.9	362	559	12	RNA-DIRECTED RNA POLYMERASE
282	2ijn	A	19.2	4	363	559	12	RNA POLYMERASE NS5B
283	4zpc	A	19.2	2.7	351	462	15	RNA-DEPENDENT RNA POLYMERASE
284	4zp9	A	19.2	2.7	349	462	15	RNA-DEPENDENT RNA POLYMERASE
285	4ju2	B	19.1	4	362	560	12	GENOME POLYPROTEIN
286	4k4y	A	19.1	2.8	352	463	14	RNA-DEPENDENT RNA POLYMERASE
287	4y3c	B	19.1	2.8	362	460	12	3D POLYMERASE
288	3mww	B	19.1	4	356	541	13	GENOME POLYPROTEIN
289	4k4z	I	19.1	2.8	351	462	14	RNA-DEPENDENT RNA POLYMERASE
290	3qgg	A	19.1	4.2	355	531	13	RNA-DIRECTED RNA POLYMERASE
291	3g86	B	19.1	4	364	558	12	RNA-DIRECTED RNA POLYMERASE
292	4k4y	M	19.1	2.8	351	463	15	RNA-DEPENDENT RNA POLYMERASE
293	3cdw	A	19.1	2.7	352	468	14	RNA-DIRECTED RNA POLYMERASE 3D-POL
294	4k4z	M	19.1	2.8	351	462	15	RNA-DEPENDENT RNA POLYMERASE
295	3h59	B	19.1	4	351	557	13	RNA-DIRECTED RNA POLYMERASE
296	4k4x	M	19.1	2.8	351	463	15	RNA-DEPENDENT RNA POLYMERASE
297	3cdu	A	19.1	2.7	352	468	14	RNA-DIRECTED RNA POLYMERASE 3D-POL
298	4k4x	A	19.1	2.8	351	463	15	RNA-DEPENDENT RNA POLYMERASE
299	3hvo	B	19.1	4.1	364	557	12	GENOME POLYPROTEIN
300	4k4z	E	19.1	2.9	352	462	14	RNA-DEPENDENT RNA POLYMERASE
301	4nlp	A	19.1	2.9	353	461	15	RNA-DIRECTED RNA POLYMERASE 3D-POL
302	4k4x	E	19.1	2.9	355	463	14	RNA-DEPENDENT RNA POLYMERASE
303	4y34	A	19.1	2.9	355	467	14	3D POLYMERASE
304	2e9z	A	19.1	2.7	362	476	11	5'-R(*CP*AP*UP*GP*GP*GP*CP*CP*C)-3'
305	6r1i	B	19.1	2.9	363	468	13	GENOME POLYPROTEIN
306	5u0c	C	19.1	3.5	410	621	12	NS5 RNA POLYMERASE DOMAIN

307	4wtl	A	19	3.8	365	553	12	RNA TEMPLATE UACC
308	1quv	A	19	4.1	365	553	13	PROTEIN (RNA-DIRECTED RNA POLYMERASE)
309	3lkh	A	19	4	363	554	12	RNA-DIRECTED RNA POLYMERASE
310	4nyz	A	19	3	362	461	12	GENOME POLYPROTEIN
311	4k4y	E	19	2.8	351	463	14	RNA-DEPENDENT RNA POLYMERASE
312	4y2a	A	19	2.7	352	466	14	3D POLYMERASE
313	1nhu	A	19	3.7	353	558	12	
314	3olb	M	19	3	356	461	15	POLYMERASE
315	2im1	A	19	2.9	358	461	15	POLIOVIRUS POLYMERASE
316	1s4f	D	19	3.5	364	575	12	RNA-DEPENDENT RNA POLYMERASE
317	2zku	D	19	4	367	558	12	GENOME POLYPROTEIN
318	4k4y	I	19	2.8	351	463	14	RNA-DEPENDENT RNA POLYMERASE
319	2who	A	19	3.9	364	531	12	RNA-DIRECTED RNA POLYMERASE
320	4k4s	E	19	3	357	462	14	RNA-DIRECTED RNA POLYMERASE 3D-POL
321	3h5u	A	19	3.9	351	559	13	RNA-DIRECTED RNA POLYMERASE
322	3vqs	A	19	4	364	553	13	RNA-DIRECTED RNA POLYMERASE
323	5m2z	F	19	13	440	884	11	NS5
324	2awz	B	19	4	365	562	13	GENOME POLYPROTEIN
325	1nb6	B	19	3.9	351	565	13	POLYPROTEIN
326	5i61	A	18.9	3	338	523	12	POTENTIAL RNA-DEPENDENT RNA POLYMERASE
327	2ijd	1	18.9	5.2	382	644	14	PICORNAIN 3C, RNA-DIRECTED RNA POLYMERASE
328	1sh0	B	18.9	3	369	503	10	RNA POLYMERASE
329	3vqs	C	18.9	3.9	364	556	12	RNA-DIRECTED RNA POLYMERASE
330	2ily	A	18.9	3	356	461	15	POLIOVIRUS POLYMERASE
331	4k4v	A	18.9	2.9	358	462	14	RNA-DIRECTED RNA POLYMERASE 3D-POL
332	4k4v	E	18.9	3	358	462	14	RNA-DIRECTED RNA POLYMERASE 3D-POL
333	3cwj	B	18.9	3.9	363	557	12	RNA-DIRECTED RNA POLYMERASE
334	3.00E+51	B	18.9	4	364	557	12	RNA-DIRECTED RNA POLYMERASE
335	3ol9	E	18.9	2.9	355	461	15	POLYMERASE
336	3ol6	A	18.9	3	358	461	15	POLYMERASE
337	4nlw	A	18.9	3	356	461	13	RNA-DIRECTED RNA POLYMERASE 3D-POL
338	1ra7	A	18.9	2.9	354	461	14	GENOME POLYPROTEIN
339	1tql	A	18.9	2.9	355	461	14	RNA-DIRECTED RNA POLYMERASE
340	1ra6	A	18.9	3	356	461	14	GENOME POLYPROTEIN
341	3h59	A	18.9	4	365	557	12	RNA-DIRECTED RNA POLYMERASE
342	2ilz	A	18.9	3	357	461	15	POLIOVIRUS POLYMERASE

343	2im3	A	18.9	2.9	355	461	15	POLIOVIRUS POLYMERASE
344	4j06	B	18.9	3.9	361	558	12	GENOME POLYPROTEIN
345	2jc1	B	18.9	4	363	545	12	RNA-DEPENDENT RNA-POLYMERASE
346	6kwr	A	18.9	3	351	453	15	RNA-DEPENDENT RNA POLYMERASE
347	5f8l	A	18.9	3	357	462	15	GENOME POLYPROTEIN
348	4ju1	B	18.8	4	362	560	12	GENOME POLYPROTEIN
349	3ol7	E	18.8	3	356	461	16	POLYMERASE
350	3hkw	B	18.8	4	363	560	12	NS5B RNA-DEPENDENT RNA POLYMERASE
351	3ol7	M	18.8	2.9	356	461	14	POLYMERASE
352	2ijf	A	18.8	2.9	354	461	14	RNA-DIRECTED RNA POLYMERASE
353	4ju4	B	18.8	4	364	558	12	GENOME POLYPROTEIN
354	6r1i	A	18.8	3	364	468	14	GENOME POLYPROTEIN
355	3ol8	I	18.8	3	358	461	15	POLYMERASE
356	3ol7	A	18.8	3	356	461	16	POLYMERASE
357	3ola	I	18.8	2.9	357	461	15	POLYMERASE
358	3ol8	A	18.8	3	356	461	16	POLYMERASE
359	4obc	A	18.8	3.8	365	554	12	RNA-DIRECTED RNA POLYMERASE
360	4lq3	A	18.8	2.9	366	495	10	RNA-DEPENDENT RNA-POLYMERASE
361	3ol7	I	18.8	2.9	357	461	16	POLYMERASE
362	3olb	I	18.8	3	357	461	15	POLYMERASE
363	3ol8	M	18.8	3	358	461	15	POLYMERASE
364	3ol8	E	18.8	3	356	461	15	POLYMERASE
365	4k4u	A	18.8	2.9	357	462	15	RNA-DIRECTED RNA POLYMERASE 3D-POL
366	3ol9	M	18.8	2.9	355	461	15	POLYMERASE
367	3ola	A	18.8	3	357	461	15	POLYMERASE
368	4k4u	E	18.8	2.9	357	462	15	RNA-DIRECTED RNA POLYMERASE 3D-POL
369	2im2	A	18.8	2.9	354	461	15	POLIOVIRUS POLYMERASE
370	4jvq	B	18.8	4	360	560	12	GENOME POLYPROTEIN
371	3ola	E	18.8	3	357	461	15	POLYMERASE
372	4nlx	A	18.8	2.9	355	461	14	RNA-DIRECTED RNA POLYMERASE 3D-POL
373	2yoj	B	18.8	4	363	558	12	RNA-DIRECTED RNA POLYMERASE
374	5ccv	C	18.8	3.5	426	853	11	RNA-DIRECTED RNA POLYMERASE NS5
375	4v0q	A	18.8	3.6	415	852	11	NS5 POLYMERASE
376	3co9	B	18.8	4	363	562	12	RNA-DIRECTED RNA POLYMERASE
377	5f8j	A	18.8	3.1	357	462	15	GENOME POLYPROTEIN
378	2qe5	A	18.8	3.9	362	559	12	RNA-DIRECTED RNA POLYMERASE
379	5u0c	H	18.8	3.5	410	623	11	NS5 RNA POLYMERASE DOMAIN
380	1xr5	A	18.8	3	355	460	17	GENOME POLYPROTEIN

381	1nb4	B	18.8	4	363	565	12	POLYPROTEIN
382	5iq6	A	18.8	3.4	396	576	11	RNA DEPENDENT RNA POLYMERASE
383	6mvq	B	18.8	4.1	364	553	12	HCV POLYMERASE
384	5f8i	A	18.8	2.9	354	462	14	GENOME POLYPROTEIN
385	5tmh	A	18.8	12.7	431	880	12	POLYPROTEIN
386	6kwq	A	18.8	3	356	462	15	RNA-DEPENDENT RNA POLYMERASE
387	5tmh	B	18.8	12.7	431	879	12	POLYPROTEIN
388	5f8h	A	18.8	3.1	356	462	14	GENOME POLYPROTEIN
389	2d3z	B	18.8	4.1	364	559	12	POLYPROTEIN
390	4ju2	A	18.7	3.9	363	560	12	GENOME POLYPROTEIN
391	4eo6	A	18.7	3.9	363	557	12	RNA-DIRECTED RNA POLYMERASE
392	4ke5	B	18.7	3.9	352	547	13	HCV POLYMERASE
393	5hn0	A	18.7	3.5	400	585	11	RNA-DIRECTED RNA POLYMERASE NS5
394	4ry7	B	18.7	4	361	565	12	HCV J4 RNA POLYMERASE (NS5B)
395	2xhu	B	18.7	4	363	563	12	RNA-DIRECTED RNA POLYMERASE
396	3cde	B	18.7	4	363	557	12	RNA-DIRECTED RNA POLYMERASE
397	4k4w	E	18.7	3	357	462	14	RNA-DIRECTED RNA POLYMERASE 3D-POL
398	4k4w	A	18.7	3	357	462	14	RNA-DIRECTED RNA POLYMERASE 3D-POL
399	4nlu	A	18.7	3	356	461	15	RNA-DIRECTED RNA POLYMERASE 3D-POL
400	5ccv	A	18.7	3.5	408	850	12	RNA-DIRECTED RNA POLYMERASE NS5
401	3hvo	A	18.7	4.1	364	557	12	GENOME POLYPROTEIN
402	5y6z	E	18.7	3	356	460	15	GENOME POLYPROTEIN
403	2brk	A	18.7	3.9	355	512	13	RNA-DIRECTED RNA POLYMERASE
404	3bsa	A	18.7	4	362	557	12	RNA-DIRECTED RNA POLYMERASE
405	4ika	A	18.7	3	353	462	14	3DPOL
406	5u0c	D	18.7	3.5	410	623	11	NS5 RNA POLYMERASE DOMAIN
407	6qwt	A	18.6	3.3	371	471	12	GENOME POLYPROTEIN
408	2ijd	2	18.6	3.8	376	644	14	PICORNAIN 3C, RNA-DIRECTED RNA POLYMERASE
409	3cvk	B	18.6	4	366	557	12	RNA-DIRECTED RNA POLYMERASE
410	5f41	A	18.6	3.5	400	585	11	GENOME POLYPROTEIN
411	2brl	A	18.6	3.9	355	512	12	RNA-DIRECTED RNA POLYMERASE
412	3cj0	B	18.6	3.9	360	562	12	RNA-DIRECTED RNA POLYMERASE
413	6j00	A	18.6	3.4	401	575	10	GENOME POLYPROTEIN
414	6h9r	A	18.6	3.6	400	558	11	GENOME POLYPROTEIN
415	2awz	A	18.6	4	365	557	12	GENOME POLYPROTEIN
416	6s2l	A	18.5	2.8	362	476	11	GENOME POLYPROTEIN
417	3q0z	A	18.5	4.2	354	551	13	RNA-DIRECTED RNA POLYMERASE
418	1nb4	A	18.5	4	363	566	12	POLYPROTEIN

419	4wtf	A	18.5	3.8	364	536	13	RNA PRIMER TEMPLATE CAAAAUUU
420	4mia	B	18.5	3.8	354	545	12	RNA-DIRECTED RNA POLYMERASE
421	1nb7	A	18.5	4	363	566	12	5'-R(*UP*UP*UP*U)-3'
422	4wtm	A	18.5	3.8	365	553	12	RNA TEMPLATE UAGG
423	4.00E+76	A	18.5	3.8	364	544	13	RNA-DIRECTED RNA POLYMERASE
424	2b43	A	18.5	3	367	501	10	NON-STRUCTURAL POLYPROTEIN
425	2qe5	C	18.5	3.9	352	559	13	RNA-DIRECTED RNA POLYMERASE
426	2ckw	A	18.5	3.2	373	487	12	RNA-DIRECTED RNA POLYMERASE
427	1xr7	A	18.5	2.7	349	460	15	GENOME POLYPROTEIN
428	4wta	A	18.4	3.8	366	535	12	RNA PRIMER TEMPLATE CAAAAUUU
429	3gyn	B	18.4	4	363	557	12	RNA-DIRECTED RNA POLYMERASE
430	4mib	B	18.4	4	363	557	12	RNA-DIRECTED RNA POLYMERASE
431	3mf5	A	18.4	3.9	362	557	12	RNA-DIRECTED RNA POLYMERASE
432	4k50	M	18.4	2.9	349	460	15	RNA POLYMERASE 3D-POL
433	4eo8	A	18.4	4	364	557	12	RNA-DIRECTED RNA POLYMERASE
434	2uut	A	18.4	3.1	373	492	12	RNA-DIRECTED RNA POLYMERASE
435	4nlq	A	18.4	3	356	461	15	RNA-DIRECTED RNA POLYMERASE 3D-POL
436	4eaw	A	18.4	3.8	349	559	13	RNA-DIRECTED RNA POLYMERASE
437	4iz0	A	18.4	4	363	559	12	RNA-DIRECTED RNA POLYMERASE
438	2qe2	B	18.4	4	330	523	14	RNA-DIRECTED RNA POLYMERASE
439	2jc1	A	18.4	4	364	549	12	RNA-DEPENDENT RNA-POLYMERASE
440	5qj1	A	18.4	3.9	355	543	12	RNA-DEPENDENT RNA POLYMERASE
441	5i3q	A	18.4	3.4	399	585	11	GENOME POLYPROTEIN
442	1xr7	B	18.4	2.8	350	460	15	GENOME POLYPROTEIN
443	1tp7	B	18.4	2.8	349	460	14	GENOME POLYPROTEIN
444	5u0c	B	18.4	3.4	413	621	12	NS5 RNA POLYMERASE DOMAIN
445	5pzp	B	18.4	4.1	358	535	13	RNA-DIRECTED RNA POLYMERASE
446	3d28	B	18.3	4	364	557	12	RNA-DIRECTED RNA POLYMERASE
447	3ske	B	18.3	3.9	360	558	12	HCV NS5B RNA_DEPENDENT RNA POLYMERASE
448	3gsz	B	18.3	4.1	364	558	12	RNA-DIRECTED RNA POLYMERASE
449	3h5s	A	18.3	3.7	354	557	12	RNA-DIRECTED RNA POLYMERASE
450	4j02	B	18.3	3.8	359	558	12	GENOME POLYPROTEIN
451	3n6n	A	18.3	3	354	462	14	RNA-DEPENDENT RNA POLYMERASE
452	4gmc	B	18.3	4.1	358	541	13	NS5B POLYMERASE
453	5ccv	H	18.3	3.8	400	767	12	RNA-DIRECTED RNA POLYMERASE NS5
454	4mk9	A	18.3	3.9	360	557	12	RNA-DIRECTED RNA POLYMERASE
455	3h5s	B	18.3	3.9	354	557	14	RNA-DIRECTED RNA POLYMERASE
456	4ih5	B	18.3	4	363	557	12	RNA-DIRECTED RNA POLYMERASE
457	4mz4	B	18.3	3.9	360	560	12	RNA-DIRECTED RNA POLYMERASE
458	2wk4	A	18.3	3.3	378	499	11	PROTEASE-POLYMERASE P70

459	4ih5	A	18.3	4	363	557	12	RNA-DIRECTED RNA POLYMERASE
460	3upi	A	18.3	4	363	563	12	RNA-DIRECTED RNA POLYMERASE
461	4nlt	A	18.3	3	356	461	14	RNA-DIRECTED RNA POLYMERASE 3D-POL
462	3bsc	B	18.3	4	364	557	12	RNA-DIRECTED RNA POLYMERASE
463	2d3u	B	18.3	3.9	362	559	12	POLYPROTEIN
464	3cj2	B	18.3	4	363	557	12	RNA-DIRECTED RNA POLYMERASE
465	3bsc	A	18.3	4.1	364	557	12	RNA-DIRECTED RNA POLYMERASE
466	3br9	B	18.3	3.8	361	557	12	RNA-DIRECTED RNA POLYMERASE
467	3cj4	B	18.3	4	363	558	12	RNA-DIRECTED RNA POLYMERASE
468	5m2z	A	18.3	13.1	439	883	11	NS5
469	2o5d	A	18.3	3.9	363	559	12	HCV
470	2qe5	D	18.3	4	363	559	12	RNA-DIRECTED RNA POLYMERASE
471	1tp7	A	18.3	2.7	348	460	14	GENOME POLYPROTEIN
472	2i1r	A	18.3	4	363	559	12	RNA-DIRECTED RNA POLYMERASE (NS5B) (P68)
473	4jty	B	18.2	3.9	360	560	13	GENOME POLYPROTEIN
474	3qgg	B	18.2	4.2	354	536	12	RNA-DIRECTED RNA POLYMERASE
475	4jy0	B	18.2	3.8	359	558	12	GENOME POLYPROTEIN
476	4ih7	A	18.2	4	363	557	12	RNA-DIRECTED RNA POLYMERASE
477	3hkw	C	18.2	4	360	560	12	NS5B RNA-DEPENDENT RNA POLYMERASE
478	4mka	A	18.2	3.8	355	557	12	RNA-DIRECTED RNA POLYMERASE
479	4mib	A	18.2	4.1	364	557	12	RNA-DIRECTED RNA POLYMERASE
480	4txs	D	18.2	4	362	544	13	POLYPROTEIN
481	4jy0	A	18.2	3.8	362	559	12	GENOME POLYPROTEIN
482	4ty9	D	18.2	3.9	360	544	13	POLYPROTEIN
483	3hky	A	18.2	4	364	564	12	RNA-DIRECTED RNA POLYMERASE
484	4mtp	C	18.2	3.5	396	585	12	RNA DEPENDENT RNA POLYMERASE
485	3cwj	A	18.2	4	363	557	12	RNA-DIRECTED RNA POLYMERASE
486	3qgh	A	18.2	4	360	559	13	RNA-DIRECTED RNA POLYMERASE
487	4j04	A	18.2	4	363	559	12	GENOME POLYPROTEIN
488	4j06	A	18.2	4	363	559	12	GENOME POLYPROTEIN
489	4j0a	B	18.2	3.9	362	558	12	GENOME POLYPROTEIN
490	2xwh	A	18.2	4.1	364	561	11	RNA DEPENDENT RNA POLYMERASE
491	4nls	A	18.2	3	357	461	15	RNA-DIRECTED RNA POLYMERASE 3D-POL
492	3fql	A	18.2	3.9	350	560	14	RNA-DIRECTED RNA POLYMERASE
493	5m2x	A	18.2	3.8	420	884	12	NS5
494	5k5m	A	18.1	3.7	403	563	11	RNA DEPENDENT RNA POLYMERASE
495	4tyb	C	18.1	4	360	545	13	POLYPROTEIN
496	3n6m	A	18.1	3.1	354	462	15	RNA-DEPENDENT RNA POLYMERASE

497	3u4r	A	18.1	4	363	558	12	RNA-DIRECTED RNA POLYMERASE
498	3mwv	B	18.1	3.9	361	558	12	GENOME POLYPROTEIN
499	3gyn	A	18.1	3.9	361	557	12	RNA-DIRECTED RNA POLYMERASE
500	4txs	C	18.1	3.9	360	544	13	POLYPROTEIN
501	3udl	D	18.1	4	362	555	12	HCV NS5B POLYMERASE
502	3udl	A	18.1	3.8	361	555	12	HCV NS5B POLYMERASE
503	3.00E+51	A	18.1	3.9	361	557	12	RNA-DIRECTED RNA POLYMERASE
504	4jtz	B	18.1	3.9	364	560	12	GENOME POLYPROTEIN
505	4khr	A	18.1	4.2	365	562	13	NS5B RNA-DEPENDENT RNA POLYMERASE
506	4ih6	A	18.1	3.9	361	557	12	RNA-DIRECTED RNA POLYMERASE
507	4j08	B	18.1	3.8	360	558	12	GENOME POLYPROTEIN
508	3gsz	A	18.1	4.1	365	558	12	RNA-DIRECTED RNA POLYMERASE
509	4ty8	D	18.1	4.1	363	546	13	POLYPROTEIN
510	4kai	A	18.1	3.8	352	552	13	HCV POLYMERASE
511	2zku	B	18.1	4	366	561	12	GENOME POLYPROTEIN
512	2xhu	A	18.1	4	362	562	12	RNA-DIRECTED RNA POLYMERASE
513	4ju1	A	18.1	3.9	363	560	12	GENOME POLYPROTEIN
514	4jty	A	18.1	3.8	361	560	12	GENOME POLYPROTEIN
515	4txs	A	18.1	4	361	544	12	POLYPROTEIN
516	3h5u	B	18.1	3.9	352	559	13	RNA-DIRECTED RNA POLYMERASE
517	4ju3	B	18.1	3.9	358	558	12	GENOME POLYPROTEIN
518	3g86	A	18.1	4	364	557	12	RNA-DIRECTED RNA POLYMERASE
519	4iz0	B	18.1	3.8	359	558	12	RNA-DIRECTED RNA POLYMERASE
520	3phe	B	18.1	4	364	558	12	HCV ENCODED NONSTRUCTURAL 5B PROTEIN
521	2d3z	A	18.1	3.9	361	560	12	POLYPROTEIN
522	1tp7	D	18.1	2.8	348	460	14	GENOME POLYPROTEIN
523	2j7w	A	18.1	3.7	396	571	11	POLYPROTEIN
524	5u0c	F	18.1	3.4	411	622	12	NS5 RNA POLYMERASE DOMAIN
525	6izx	A	18.1	3.5	407	581	12	GENOME POLYPROTEIN
526	4mk8	A	18	3.8	355	557	12	RNA-DIRECTED RNA POLYMERASE
527	3udl	B	18	3.9	362	555	12	HCV NS5B POLYMERASE
528	4tya	A	18	4	362	544	12	POLYPROTEIN
529	3mf5	B	18	4.1	365	557	12	RNA-DIRECTED RNA POLYMERASE
530	4j0a	A	18	4	363	559	12	GENOME POLYPROTEIN
531	3fqk	B	18	3.8	355	557	12	RNA-DIRECTED RNA POLYMERASE
532	4ry5	A	18	4	363	565	12	HCV J4 RNA POLYMERASE (NS5B)
533	4jy1	A	18	4	363	559	12	GENOME POLYPROTEIN
534	4jy1	B	18	3.9	359	558	12	GENOME POLYPROTEIN
535	4oow	B	18	4	356	538	13	RNA-DIRECTED RNA POLYMERASE
536	3tyq	B	18	4	362	558	12	RNA-DIRECTED RNA POLYMERASE

537	4kb7	A	18	3.8	350	545	13	HCV POLYMERASE
538	4ih7	B	18	4	363	557	12	RNA-DIRECTED RNA POLYMERASE
539	2xhv	A	18	4	360	563	12	RNA-DIRECTED RNA POLYMERASE
540	4mz4	A	18	4	363	563	12	RNA-DIRECTED RNA POLYMERASE
541	3mwv	A	18	4	362	559	12	GENOME POLYPROTEIN
542	3cj3	A	18	3.9	361	560	12	RNA-DIRECTED RNA POLYMERASE
543	2gc8	A	18	4.1	366	557	12	RNA-DIRECTED RNA POLYMERASE
544	3fqk	A	17.9	3.9	352	557	13	RNA-DIRECTED RNA POLYMERASE
545	4jtz	A	17.9	3.9	363	560	12	GENOME POLYPROTEIN
546	3phe	C	17.9	4	365	558	13	HCV ENCODED NONSTRUCTURAL 5B PROTEIN
547	4jvq	A	17.9	3.9	363	560	12	GENOME POLYPROTEIN
548	4ty9	C	17.9	3.9	360	544	12	POLYPROTEIN
549	4ju4	A	17.9	3.9	363	559	12	GENOME POLYPROTEIN
550	4eo6	B	17.9	4	364	560	12	RNA-DIRECTED RNA POLYMERASE
551	4wte	A	17.9	3.8	366	535	12	RNA PRIMER TEMPLATE ACAAAUUU
552	3phe	A	17.9	3.9	365	558	12	HCV ENCODED NONSTRUCTURAL 5B PROTEIN
553	5yf6	A	17.9	3.3	422	646	13	RDRP CATALYTIC
554	3d5m	B	17.9	4	365	557	12	RNA-DIRECTED RNA POLYMERASE
555	4tya	C	17.9	3.9	359	544	13	POLYPROTEIN
556	3ska	A	17.9	3.9	352	563	14	HCV NS5B RNA_DEPENDENT RNA POLYMERASE
557	4jtw	A	17.9	3.8	362	560	12	GENOME POLYPROTEIN
558	3tyq	A	17.9	4	363	563	12	RNA-DIRECTED RNA POLYMERASE
559	5dto	A	17.9	3.6	418	853	11	NS5
560	4v0r	A	17.9	3.6	417	853	11	NS5 POLYMERASE
561	4hhj	A	17.9	3.5	399	588	11	NON-STRUCTURAL PROTEIN 5
562	3qgi	A	17.9	4.3	367	561	13	RNA-DIRECTED RNA POLYMERASE
563	2hwi	A	17.9	4	364	559	12	RNA-DIRECTED RNA POLYMERASE (NS5B) (P68)
564	5trh	A	17.9	4.2	354	550	13	NS5B RNA-DEPENDENT RNA POLYMERASE
565	6ae7	A	17.9	5.3	405	642	13	RDRP CATALYTIC
566	3ciz	B	17.9	4	363	563	12	RNA-DIRECTED RNA POLYMERASE
567	1c2p	B	17.8	4	364	561	12	RNA-DEPENDENT RNA POLYMERASE
568	4j08	A	17.8	3.8	361	559	12	GENOME POLYPROTEIN
569	4dru	A	17.8	4	355	549	12	RNA-DIRECTED RNA POLYMERASE
570	3skh	B	17.8	4	361	558	12	HCV NS5B RNA_DEPENDENT RNA POLYMERASE
571	4jtw	B	17.8	4	361	560	12	GENOME POLYPROTEIN
572	3udl	C	17.8	3.8	360	555	12	HCV NS5B POLYMERASE
573	3vws	A	17.8	3.6	409	592	11	NON-STRUCTURAL PROTEIN 5

574	4c11	A	17.8	3.5	400	593	11	DENGUE VIRUS TYPE 3 RNA DEPENDENT RNA POLYMERASE
575	3cj5	A	17.8	3.8	355	562	12	RNA-DIRECTED RNA POLYMERASE
576	6mvq	A	17.8	3.9	352	552	13	HCV POLYMERASE
577	3cj3	B	17.8	4	362	562	12	RNA-DIRECTED RNA POLYMERASE
578	4ju3	A	17.7	4	363	559	12	GENOME POLYPROTEIN
579	3gnv	B	17.7	4.1	361	553	12	RNA-DIRECTED RNA POLYMERASE
580	4ry7	A	17.7	3.9	363	565	12	HCV J4 RNA POLYMERASE (NS5B)
581	4jjs	A	17.7	4	363	559	12	GENOME POLYPROTEIN
582	5f3t	A	17.7	3.5	400	585	11	RNA-DEPENDENT RNA POLYMERASE
583	2gir	A	17.7	4	362	557	12	RNA-DIRECTED RNA POLYMERASE
584	5pzk	A	17.7	3.8	341	541	14	RNA-DIRECTED RNA POLYMERASE
585	5u0c	A	17.7	3.4	412	624	12	NS5 RNA POLYMERASE DOMAIN
586	5hmx	A	17.6	3.5	398	590	11	RNA-DIRECTED RNA POLYMERASE NS5
587	4jju	B	17.6	4	362	558	12	GENOME POLYPROTEIN
588	5f3z	A	17.6	3.5	399	585	11	GENOME POLYPROTEIN
589	4mk7	A	17.6	4.1	365	557	12	RNA-DIRECTED RNA POLYMERASE
590	1rdr	A	17.6	2.7	273	316	14	POLIOVIRUS 3D POLYMERASE
591	3ske	A	17.5	3.9	352	563	13	HCV NS5B RNA_DEPENDENT RNA POLYMERASE
592	4tyb	D	17.5	3.9	360	545	13	POLYPROTEIN
593	3gnw	A	17.5	4	363	560	12	RNA-DIRECTED RNA POLYMERASE
594	4j04	B	17.5	3.9	361	558	12	GENOME POLYPROTEIN
595	4ty8	A	17.5	3.9	360	544	12	POLYPROTEIN
596	3gol	B	17.5	4	350	559	13	RNA-DIRECTED RNA POLYMERASE
597	5tfr	A	17.4	12.7	431	883	12	GENOME POLYPROTEIN
598	4jjs	B	17.4	4	364	558	12	GENOME POLYPROTEIN
599	4mk7	B	17.4	4	365	559	12	RNA-DIRECTED RNA POLYMERASE
600	4ty9	A	17.4	3.8	359	544	12	POLYPROTEIN
601	4aex	B	17.4	4.1	364	564	12	RNA-DIRECTED RNA POLYMERASE
602	4ry6	B	17.4	4	362	564	12	HCV J4 RNA POLYMERASE (NS5B)
603	4mtp	B	17.4	3.5	397	591	12	RNA DEPENDENT RNA POLYMERASE
604	4adp	A	17.4	4	364	564	11	RNA-DIRECTED RNA POLYMERASE
605	3br9	A	17.4	3.8	351	557	13	RNA-DIRECTED RNA POLYMERASE
606	5pzk	B	17.4	4	343	542	15	RNA-DIRECTED RNA POLYMERASE
607	5m2x	F	17.4	13.2	434	884	12	NS5
608	3cso	A	17.3	4	364	561	12	RNA-DIRECTED RNA POLYMERASE
609	3mww	A	17.3	4	363	559	12	GENOME POLYPROTEIN
610	2xhv	B	17.3	4	362	562	12	RNA-DIRECTED RNA POLYMERASE
611	4ju7	B	17.3	4	362	558	12	GENOME POLYPROTEIN
612	4ju6	B	17.3	4	362	558	12	GENOME POLYPROTEIN
613	6mvo	B	17.3	4.1	352	562	14	RNA-DIRECTED RNA POLYMERASE

614	2d41	B	17.3	3.9	362	558	12	POLYPROTEIN
615	1nb7	B	17.3	3.9	363	565	12	5'-R(*UP*UP*UP*U)-3'
616	3gol	A	17.2	4.1	362	557	12	RNA-DIRECTED RNA POLYMERASE
617	3hkw	A	17.2	4.1	364	561	12	NS5B RNA-DEPENDENT RNA POLYMERASE
618	3igv	A	17.2	4	363	557	12	RNA-DIRECTED RNA POLYMERASE
619	4jju	A	17.2	3.9	363	559	12	GENOME POLYPROTEIN
620	6i7p	A	17.2	4	422	884	10	NS5
621	5czb	A	17.2	4	361	555	12	NS5B
622	5u0c	G	17.2	3.5	411	624	12	NS5 RNA POLYMERASE DOMAIN
623	4wtc	A	17.1	3.8	366	534	12	RNA PRIMER TEMPLATE AGAAAUUU
624	3uph	A	17.1	4	363	563	12	RNA-DIRECTED RNA POLYMERASE
625	4khm	B	17.1	3.9	350	562	14	HCV POLYMERASE
626	2xi2	B	17.1	4	363	559	12	RNA-DIRECTED RNA POLYMERASE
627	4mtp	A	17.1	3.6	397	591	12	RNA DEPENDENT RNA POLYMERASE
628	4mk8	B	17.1	3.8	361	557	12	RNA-DIRECTED RNA POLYMERASE
629	4nld	A	17.1	3.7	340	500	12	RNA-DIRECTED RNA POLYMERASE
630	4hdg	A	17.1	3.6	410	612	12	POLYPROTEIN
631	5ccv	D	17.1	3.5	414	849	11	RNA-DIRECTED RNA POLYMERASE NS5
632	4hdh	B	17.1	3.5	406	613	13	POLYPROTEIN
633	5ccv	G	17.1	3.5	413	848	11	RNA-DIRECTED RNA POLYMERASE NS5
634	4hdg	B	17	3.6	411	612	12	POLYPROTEIN
635	5tri	A	16.9	4.1	354	542	13	NS5B RNA-DEPENDENT RNA POLYMERASE
636	5zqk	A	16.8	14	434	875	10	NON STRUCTURAL PROTEIN 5
637	2qe2	A	16.8	3.7	330	521	14	RNA-DIRECTED RNA POLYMERASE
638	4kbi	A	16.7	3.9	351	544	13	HCV POLYMERASE
639	5m2z	B	16.7	12.5	443	883	10	NS5
640	1c2p	A	16.6	4	363	558	12	RNA-DEPENDENT RNA POLYMERASE
641	4hdh	A	16.6	3.6	410	613	12	POLYPROTEIN
642	3igv	B	16.5	4	364	557	12	RNA-DIRECTED RNA POLYMERASE
643	4mk9	B	16.4	3.9	363	557	12	RNA-DIRECTED RNA POLYMERASE
644	4ju7	A	16.4	4	363	559	12	GENOME POLYPROTEIN
645	4ju6	A	16.4	4	363	559	12	GENOME POLYPROTEIN
646	4c11	B	16.4	3.5	403	603	11	DENGUE VIRUS TYPE 3 RNA DEPENDENT RNA POLYMERASE
647	5pzo	A	16.4	4.1	355	550	13	RNA-DIRECTED RNA POLYMERASE
648	6qsn	B	16.4	5	415	885	11	GENOME POLYPROTEIN
649	6izy	A	16.4	3.5	412	586	11	GENOME POLYPROTEIN
650	5hmy	A	16.1	3.5	400	591	11	RNA-DIRECTED RNA POLYMERASE NS5
651	4ty8	C	16.1	3.8	359	545	12	POLYPROTEIN
652	2hfz	A	16	3.8	404	608	12	RNA-DIRECTED RNA POLYMERASE(NS5)

653	1nhu	B	15.8	4	364	557	12	
654	5zqk	B	15.7	4.9	424	876	11	NON STRUCTURAL PROTEIN 5
655	5m2x	E	15.7	13.2	435	884	12	NS5
656	1raj	A	15.6	2.6	230	257	15	GENOME POLYPROTEIN
657	5m2z	C	15.6	13.5	441	884	10	NS5
658	5twm	A	15.5	4.1	351	559	13	NS5B RNA-DEPENDENT RNA POLYMERASE
659	6mvk	A	15.5	4	363	550	12	HCV POLYMERASE
660	2j7u	A	15.4	3.5	396	573	10	RNA DEPENDENT RNA POLYMERASE
661	5i3p	A	15.4	3.5	399	585	11	GENOME POLYPROTEIN
662	2d41	A	15.4	3.9	363	558	12	POLYPROTEIN
663	3h98	B	15.3	4	362	559	12	RNA-DIRECTED RNA POLYMERASE
664	4e7a	A	15.3	3.8	364	536	13	5'-R(*CP*AP*UP*GP*GP*C)-D(P*(DOC))-3'
665	5m2z	D	15.2	4	426	883	10	NS5
666	4xha	A	15.1	3.4	360	663	12	RNA-DEPENDENT RNA POLYMERASE
667	4wtg	A	15	3.8	366	535	12	RNA PRIMER TEMPLATE CAAAAUUU
668	5trh	B	14.9	4	350	542	13	NS5B RNA-DEPENDENT RNA POLYMERASE
669	6mvp	A	14.9	3.9	353	548	14	GENOME POLYPROTEIN
670	5u0b	A	14.9	12.7	429	884	10	GENOME POLYPROTEIN
671	4.00E+78	A	14.8	3.7	364	538	13	5'-R(*U*AP*CP*CP*GP*(GDO))-3'
672	4wt9	A	14.8	3.8	364	544	12	RNA-DIRECTED RNA POLYMERASE
673	6i7p	D	14.8	13	440	884	10	NS5
674	5i61	B	14.7	3.1	334	510	12	POTENTIAL RNA-DEPENDENT RNA POLYMERASE
675	5pzo	B	14.7	4	355	529	13	RNA-DIRECTED RNA POLYMERASE
676	4kbi	B	14.6	4.1	366	545	12	HCV POLYMERASE
677	2yoj	A	14.5	4	362	563	12	RNA-DIRECTED RNA POLYMERASE
678	4ty8	B	14.4	4	362	547	12	POLYPROTEIN
679	4tyb	B	14.4	4	362	544	12	POLYPROTEIN
680	4txs	B	14.4	3.9	361	544	12	POLYPROTEIN
681	4ih6	B	14.4	4	364	557	12	RNA-DIRECTED RNA POLYMERASE
682	4tya	D	14.4	4	360	544	13	POLYPROTEIN
683	3lkh	B	14.4	3.8	362	558	12	RNA-DIRECTED RNA POLYMERASE
684	4ty9	B	14.4	3.9	361	544	12	POLYPROTEIN
685	4mka	B	14.4	4	364	557	12	RNA-DIRECTED RNA POLYMERASE
686	1nhv	A	14.4	3.8	363	558	12	
687	5cyr	A	14.4	3.4	359	662	12	RNA-DEPENDENT RNA POLYMERASE
688	6mvp	B	14.4	4.1	366	550	12	GENOME POLYPROTEIN
689	3h2l	B	14.3	4	363	557	12	NS5B POLYMERASE
690	4tya	B	14.3	4.1	362	544	13	POLYPROTEIN
691	4wti	A	14.3	3.8	366	553	12	RNA TEMPLATE ACGG

692	3h2l	A	14.3	4	363	557	12	NS5B POLYMERASE
693	3uph	B	14.3	3.8	359	558	12	RNA-DIRECTED RNA POLYMERASE
694	3tyv	B	14.3	3.9	359	558	12	RNA-DIRECTED RNA POLYMERASE
695	4tyb	A	14.3	3.9	359	545	12	POLYPROTEIN
696	5pzn	B	14.3	4	354	540	12	RNA-DIRECTED RNA POLYMERASE
697	5twn	B	14.3	4.1	354	536	12	NS5B RNA- DEPENDENT RNA POLYMERASE
698	2d3u	A	14.3	3.8	362	558	12	POLYPROTEIN
699	2giq	A	14.3	4	363	557	12	RNA-DIRECTED RNA POLYMERASE
700	4eo8	B	14.2	3.9	361	560	12	RNA-DIRECTED RNA POLYMERASE
701	3d28	A	14.2	4.1	363	557	12	RNA-DIRECTED RNA POLYMERASE
702	5pzn	A	14.2	4.2	355	545	13	RNA-DIRECTED RNA POLYMERASE
703	2ijn	B	14.2	4.1	364	561	12	RNA POLYMERASE NS5B
704	5pzp	A	14.2	4.2	355	545	12	RNA-DIRECTED RNA POLYMERASE
705	1uvn	C	14.2	4.2	343	664	12	RNA-DEPENDENT RNA POLYMERASE
706	1yv2	A	14.2	3.9	362	548	12	RNA DEPENDENT RNA POLYMERASE
707	5w2e	B	14.2	4	362	559	12	GENOME POLYPROTEIN
708	5trj	B	14.2	4.1	355	543	13	RNA-DEPENDENT RNA POLYMERASE
709	2giq	B	14.2	4	364	557	12	RNA-DIRECTED RNA POLYMERASE
710	5w2e	A	14.2	4	365	559	12	GENOME POLYPROTEIN
711	4ry6	A	14.1	4	363	565	12	HCV J4 RNA POLYMERASE (NS5B)
712	3cso	B	14.1	3.9	361	560	12	RNA-DIRECTED RNA POLYMERASE
713	3ciz	A	14.1	4	364	562	12	RNA-DIRECTED RNA POLYMERASE
714	4gmc	A	14.1	4	363	559	12	NS5B POLYMERASE
715	3co9	A	14.1	3.9	361	562	12	RNA-DIRECTED RNA POLYMERASE
716	2fvc	B	14.1	3.9	363	563	12	POLYPROTEIN
717	5pzm	B	14.1	4.2	355	545	13	RNA-DIRECTED RNA POLYMERASE
718	5trk	A	14.1	4.2	354	551	13	GENOME POLYPROTEIN
719	1uvn	A	14.1	4.2	343	664	12	RNA-DEPENDENT RNA POLYMERASE
720	2xi3	B	14	4.1	364	562	13	RNA-DIRECTED RNA POLYMERASE
721	1nb6	A	14	4	363	566	12	POLYPROTEIN
722	5pzl	A	14	4.1	352	551	13	RNA-DIRECTED RNA POLYMERASE
723	5pzm	A	14	4.2	354	551	13	RNA-DIRECTED RNA POLYMERASE
724	4mtp	D	13.9	3.5	377	546	10	RNA DEPENDENT RNA POLYMERASE
725	4a8y	C	13.9	4	340	640	12	RNA-DIRECTED RNA POLYMERASE
726	2jlf	B	13.9	4.1	346	661	12	RNA-DIRECTED RNA POLYMERASE
727	4b02	A	13.8	3.9	339	664	11	RNA-DIRECTED RNA POLYMERASE
728	1uvl	A	13.8	4.2	345	664	12	RNA-DEPENDENT RNA POLYMERASE
729	1uvl	C	13.7	3.9	338	664	11	RNA-DEPENDENT RNA POLYMERASE
730	1hi8	B	13.7	4.1	340	664	11	P2 PROTEIN
731	2hcs	A	13.7	3.8	346	486	13	RNA-DIRECTED RNA POLYMERASE (NS5)
732	1uvl	E	13.6	3.9	339	664	11	RNA-DEPENDENT RNA POLYMERASE

733	4a8w	A	13.6	4.2	347	664	12	RNA-DIRECTED RNA POLYMERASE
734	5m2x	D	13.6	12.5	438	884	11	NS5
735	2hcn	A	13.5	3.8	344	486	12	RNA-DIRECTED RNA POLYMERASE (NS5)
736	4a8s	B	13.5	4.2	344	664	12	RNA-DIRECTED RNA POLYMERASE
737	1uvi	C	13.5	4.2	346	664	12	RNA-DEPENDENT RNA POLYMERASE
738	1hhs	A	13.4	4.2	345	664	12	RNA-DIRECTED RNA POLYMERASE
739	4a8m	R	13.4	4.2	347	664	12	RNA-DIRECTED RNA POLYMERASE
740	1uvj	A	13.4	4.1	344	664	12	P2 PROTEIN
741	4a8s	C	13.4	4.1	340	646	12	RNA-DIRECTED RNA POLYMERASE
742	4a8k	A	13.3	4.2	348	664	12	RNA-DIRECTED RNA POLYMERASE
743	4a8k	C	13.3	4.2	344	664	12	RNA-DIRECTED RNA POLYMERASE
744	5qj0	A	13.3	3.9	349	559	12	RNA-DEPENDENT RNA POLYMERASE
745	1hht	Q	13.3	4.1	341	664	12	P2 PROTEIN
746	1hht	P	13.3	4.2	343	664	12	P2 PROTEIN
747	1hht	R	13.3	4.2	344	664	12	P2 PROTEIN
748	2jl9	A	13.3	4.1	343	661	12	RNA-DIRECTED RNA POLYMERASE
749	4a8o	A	13.2	4.2	348	664	12	RNA-DIRECTED RNA POLYMERASE
750	5ccv	E	13.2	3.7	430	853	11	RNA-DIRECTED RNA POLYMERASE NS5
751	4a8q	C	13.1	4	337	647	12	RNA-DIRECTED RNA POLYMERASE
752	1hhs	C	13.1	4	335	664	10	RNA-DIRECTED RNA POLYMERASE
753	1uvm	A	13.1	4	339	664	11	RNA-DEPENDENT RNA POLYMERASE
754	1uvn	E	13.1	4.2	344	664	12	RNA-DEPENDENT RNA POLYMERASE
755	4ieg	D	13.1	4	324	643	10	RNA-DEPENDENT RNA POLYMERASE P2
756	1hhs	B	13	4.1	337	664	12	RNA-DIRECTED RNA POLYMERASE
757	1wac	C	13	4	338	662	12	P2 PROTEIN
758	5m2x	C	13	12.5	437	884	11	NS5
759	1wac	A	13	4	338	662	12	P2 PROTEIN
760	1wac	B	13	3.9	335	662	12	P2 PROTEIN
761	1uvj	B	13	4	340	664	10	P2 PROTEIN
762	4xhi	A	12.9	3.4	361	663	12	RNA-DEPENDENT RNA POLYMERASE
763	1uvm	C	12.9	4.2	346	664	12	RNA-DEPENDENT RNA POLYMERASE
764	6qpg	H	12.9	4.3	310	667	10	POLYMERASE ACIDIC PROTEIN
765	5u04	A	12.9	3.4	331	478	12	ZIKA VIRUS NS5 RDRP
766	6ty9	A	12.8	6.5	343	1208	8	RNA-DEPENDENT RNA POLYMERASE
767	4a8f	C	12.8	4	343	664	11	RNA-DIRECTED RNA POLYMERASE
768	4xhi	B	12.8	3.4	358	664	12	RNA-DEPENDENT RNA POLYMERASE
769	5tit	A	12.8	3.4	330	478	12	ZIKA VIRUS NS5 RDRP
770	4ieg	A	12.8	4.1	329	649	10	RNA-DEPENDENT RNA POLYMERASE P2
771	4a8m	Q	12.7	4.2	345	664	12	RNA-DIRECTED RNA POLYMERASE
772	4a8y	B	12.7	4.1	340	664	11	RNA-DIRECTED RNA POLYMERASE
773	1uvj	C	12.7	4.1	343	664	12	P2 PROTEIN

774	6tz0	A	12.6	6.8	344	1208	8	RNA-DEPENDENT RNA POLYMERASE
775	6qnw	B	12.6	4.3	306	679	12	POLYMERASE ACIDIC PROTEIN
776	6u1x	A	12.6	4.2	338	2059	7	RNA-DIRECTED RNA POLYMERASE L
777	6qnw	E	12.5	4.4	307	665	11	POLYMERASE ACIDIC PROTEIN
778	2jl9	C	12.5	4.1	342	655	12	RNA-DIRECTED RNA POLYMERASE
779	4gzk	A	12.4	3.9	321	650	11	RNA-DEPENDENT RNA POLYMERASE P2
780	5fj6	A	12.4	4.2	337	664	11	RNA-DIRECTED RNA POLYMERASE
781	6qnw	H	12.3	4.3	300	676	10	POLYMERASE ACIDIC PROTEIN
782	5u0b	B	12.2	4.9	425	884	12	GENOME POLYPROTEIN
783	5cx6	B	12.1	3.4	359	665	12	RNA-DEPENDENT RNA POLYMERASE
784	5cx6	A	12.1	3.3	358	663	12	RNA-DEPENDENT RNA POLYMERASE
785	4xha	B	12.1	3.3	360	662	12	RNA-DEPENDENT RNA POLYMERASE
786	6ar3	A	12.1	4.3	294	418	10	GSI-IIC RT
787	5fj7	C	12	4.1	341	664	11	MAJOR INNER PROTEIN P1
788	5cyr	B	12	3.5	360	664	12	RNA-DEPENDENT RNA POLYMERASE
789	6qcv	B	11.9	4.3	305	746	10	POLYMERASE ACIDIC PROTEIN
790	6kur	B	11.9	4.2	301	705	13	POLYMERASE 3
791	2r7t	A	11.8	3.8	314	1073	10	RNA (5'-R(*UP*GP*UP*GP*AP*AP*CP*C)-3')
792	2yi8	A	11.8	3.4	350	771	11	RNA-DIRECTED RNA POLYMERASE
793	6ogz	A	11.8	6.7	334	1082	10	RNA (5'-
794	6qpg	B	11.8	4.4	310	656	9	POLYMERASE ACIDIC PROTEIN
795	6kut	B	11.8	4.3	307	696	13	POLYMERASE 3
796	6qcw	B	11.8	4.3	309	745	10	POLYMERASE ACIDIC PROTEIN
797	2r7w	A	11.7	3.8	314	1073	10	RNA (5'-R(*UP*GP*UP*GP*AP*CP*C)-3')
798	6ar3	D	11.7	4.2	285	419	9	GSI-IIC RT
799	6qpg	E	11.7	4.3	311	666	11	POLYMERASE ACIDIC PROTEIN
800	2yi9	A	11.7	3.4	352	771	10	RNA-DIRECTED RNA POLYMERASE
801	5m2x	B	11.7	4.1	421	883	12	NS5
802	6i7p	F	11.7	13.2	434	884	12	NS5
803	4r71	B	11.6	4.3	263	556	10	ELONGATION FACTOR TS, ELONGATION FACTOR TU
804	6i7p	B	11.6	13.3	441	883	11	NS5
805	2r7x	B	11.6	6.6	320	1073	10	RNA (5'-R(*UP*GP*UP*GP*AP*CP*C)-3')
806	6ju2	B	11.5	4.2	307	703	14	POLYMERASE 3
807	4q7j	C	11.5	4.4	279	547	11	ELONGATION FACTOR TS
808	6i7p	C	11.5	12.7	437	884	11	NS5
809	2r72	A	11.5	3.5	352	765	11	INFECTIOUS BURSAL DISEASE VIRUS VP1 POLYMERASE
810	4wsa	B	11.5	4.1	302	733	11	INFLUENZA B VRNA PROMOTER 3' END
811	6qct	B	11.5	4.1	300	735	10	POLYMERASE ACIDIC PROTEIN
812	6qpf	B	11.4	4.2	307	685	10	POLYMERASE ACIDIC PROTEIN
813	6qcs	B	11.4	4.1	298	750	10	POLYMERASE ACIDIC PROTEIN

814	4r71	D	11.4	4.7	273	553	10	ELONGATION FACTOR TS, ELONGATION FACTOR TU
815	3vnv	A	11.4	4.6	272	1203	10	
816	5m2z	E	11.4	13.1	441	884	11	NS5
817	3avw	A	11.3	4.3	269	1203	10	
818	3agp	A	11.3	5.5	271	1202	10	
819	2yi9	C	11.2	3.3	351	771	10	RNA-DIRECTED RNA POLYMERASE
820	6qsn	A	11.2	5.7	421	883	11	GENOME POLYPROTEIN
821	3avt	A	11.1	5.7	271	1203	9	
822	6abe	B	11.1	4.3	306	702	13	POLYMERASE 3
823	5irf	A	11.1	4.1	232	283	8	RETRON-TYPE REVERSE TRANSCRIPTASE
824	5irf	D	11.1	4.1	231	290	8	RETRON-TYPE REVERSE TRANSCRIPTASE
825	2qj1	A	11.1	3.4	351	767	10	INFECTIOUS BURSAL DISEASE VIRUS VP1 POLYMERASE
826	4ieg	C	11.1	4.1	327	650	10	RNA-DEPENDENT RNA POLYMERASE P2
827	5hhj	B	11.1	4.2	232	293	8	RETRON-TYPE REVERSE TRANSCRIPTASE
828	3agq	A	11.1	4.6	276	1199	9	
829	5irf	C	11.1	4.1	231	282	8	RETRON-TYPE REVERSE TRANSCRIPTASE
830	5irg	B	11.1	4.2	232	288	8	RETRON-TYPE REVERSE TRANSCRIPTASE
831	5fmz	E	11.1	4.3	306	733	11	POLYMERASE ACIDIC PROTEIN
832	6v85	A	11	4.1	303	1901	9	RNA-DIRECTED RNA POLYMERASE L
833	5irg	A	11	4.1	231	286	8	RETRON-TYPE REVERSE TRANSCRIPTASE
834	5irg	D	11	4.2	230	286	8	RETRON-TYPE REVERSE TRANSCRIPTASE
835	6ueb	A	11	4.1	339	2099	8	LARGE STRUCTURAL PROTEIN
836	5irg	C	11	4.2	230	284	8	RETRON-TYPE REVERSE TRANSCRIPTASE
837	5hhl	G	11	4.3	235	289	9	RETRON-TYPE REVERSE TRANSCRIPTASE
838	5hhj	A	11	4.1	232	296	8	RETRON-TYPE REVERSE TRANSCRIPTASE
839	6kuk	B	11	4.2	303	701	13	POLYMERASE 3
840	5hhk	B	11	4.2	232	292	8	RETRON-TYPE REVERSE TRANSCRIPTASE
841	3vnu	A	10.9	4.6	272	1203	10	
842	4fwt	A	10.9	5.9	285	1203	10	
843	5hhl	B	10.9	4.3	234	290	8	RETRON-TYPE REVERSE TRANSCRIPTASE
844	5hhk	A	10.9	4.3	233	294	8	RETRON-TYPE REVERSE TRANSCRIPTASE
845	5hhl	E	10.9	4.2	231	289	9	RETRON-TYPE REVERSE TRANSCRIPTASE
846	5fmz	B	10.9	4.3	303	708	10	POLYMERASE ACIDIC PROTEIN
847	5hhl	A	10.9	4.3	236	292	8	RETRON-TYPE REVERSE TRANSCRIPTASE
848	5hhl	D	10.9	4.3	232	289	8	RETRON-TYPE REVERSE TRANSCRIPTASE
849	6j6g	A	10.9	3.9	333	1913	8	PRE-MRNA-SPLICING FACTOR 8
850	5hhl	F	10.8	4.4	230	283	8	RETRON-TYPE REVERSE TRANSCRIPTASE
851	5hhl	C	10.8	4.3	231	289	8	RETRON-TYPE REVERSE TRANSCRIPTASE
852	5hhl	H	10.8	4.3	231	287	8	RETRON-TYPE REVERSE TRANSCRIPTASE
853	5m3j	B	10.8	4.3	307	733	10	POLYMERASE ACIDIC PROTEIN

854	6evj	B	10.7	4.2	307	752	10	POLYMERASE ACIDIC PROTEIN
855	6qpf	H	10.7	4.3	304	673	10	POLYMERASE ACIDIC PROTEIN
856	2yib	D	10.7	3.5	353	761	10	RNA-DIRECTED RNA POLYMERASE
857	6qpf	E	10.7	4.4	306	661	10	POLYMERASE ACIDIC PROTEIN
858	5msg	B	10.7	4.4	303	746	12	POLYMERASE ACIDIC PROTEIN
859	6uen	A	10.6	4	307	1392	12	RNA-DIRECTED RNA POLYMERASE L
860	2r7u	A	10.6	3.8	315	1073	10	RNA (5'-R(*AP*A*AP*AP*GP*CP*C)-3')
861	2yi8	C	10.6	3.3	347	771	11	RNA-DIRECTED RNA POLYMERASE
862	6kuj	B	10.6	4.2	306	705	13	POLYMERASE 3
863	5irf	B	10.6	4.4	231	287	8	RETRON-TYPE REVERSE TRANSCRIPTASE
864	3zed	C	10.5	3.4	352	765	11	RNA-DIRECTED RNA POLYMERASE
865	6ju3	B	10.5	4.2	300	702	12	POLYMERASE 3
866	5d98	B	10.5	4.4	298	711	12	POLYMERASE ACIDIC PROTEIN
867	4wrt	B	10.5	4.4	306	742	11	INFLUENZA VIRUS POLYMERASE VRNA PROMOTER 3' END
868	6f5o	B	10.5	4.1	301	733	9	POLYMERASE ACIDIC PROTEIN
869	6kv5	B	10.5	4.3	306	696	12	POLYMERASE 3
870	6qcx	B	10.5	4.3	304	747	10	POLYMERASE ACIDIC PROTEIN
871	5lj3	A	10.5	4	338	1922	7	U5 SNRNA (SMALL NUCLEAR RNA)
872	2yi9	E	10.4	3.4	351	771	10	RNA-DIRECTED RNA POLYMERASE
873	2yi8	E	10.4	3.4	351	771	11	RNA-DIRECTED RNA POLYMERASE
874	4q7j	G	10.4	4.6	272	545	11	ELONGATION FACTOR TS
875	2yi8	D	10.4	3.4	351	771	11	RNA-DIRECTED RNA POLYMERASE
876	6ar1	D	10.4	4.3	291	415	10	GSI-IIC RT
877	5a22	A	10.3	4.2	338	2002	7	VESICULAR STOMATITIS VIRUS L POLYMERASE
878	4wsb	B	10.3	4.1	299	745	10	POLYMERASE PA
879	1uon	A	10.3	6.6	337	1264	9	MINOR CORE PROTEIN LAMBDA 3
880	6ogy	A	10.3	7.3	325	1053	8	RNA-DEPENDENT RNA POLYMERASE OF ROTAVIRUS A
881	6ar1	A	10.3	4.1	286	417	10	GSI-IIC RT
882	6f5p	B	10.3	4.5	305	710	13	POLYMERASE ACIDIC PROTEIN
883	6abg	B	10.2	4.3	313	695	15	POLYMERASE 3
884	2r70	A	10.2	3.4	351	752	11	INFECTIOUS BURSAL VIRUS VP1 POLYMERASE
885	5amq	A	10.1	6.8	366	1666	8	RNA POLYMERASE L
886	5d9a	B	10.1	4.4	299	711	13	POLYMERASE ACIDIC PROTEIN
887	5gap	A	10	3.9	334	1349	7	U4 SNRNA, 5' REGION, NUCLEOTIDES 1-67
888	2r7q	A	10	3.9	316	1072	9	RNA-DEPENDENT RNA POLYMERASE
889	6ab7	B	10	4.2	300	702	14	POLYMERASE 3
890	6evk	B	10	4.1	305	748	10	POLYMERASE ACIDIC PROTEIN
891	2yia	D	9.9	3.4	352	790	11	RNA-DIRECTED RNA POLYMERASE

892	6abf	B	9.9	4.2	307	702	13	
893	6abb	B	9.9	4.5	301	702	13	
894	2pgg	A	9.9	3.4	351	765	11	RNA-DIRECTED RNA POLYMERASE
895	6evj	E	9.9	4.2	307	749	10	POLYMERASE ACIDIC PROTEIN
896	6kuv	B	9.9	4.2	307	705	14	POLYMERASE 3
897	2yib	A	9.8	3.4	350	789	11	RNA-DIRECTED RNA POLYMERASE
898	3avy	A	9.8	5.7	267	1203	10	
899	2yib	B	9.8	3.4	350	789	11	RNA-DIRECTED RNA POLYMERASE
900	1hi1	B	9.8	4.2	342	664	12	P2 PROTEIN
901	2yia	E	9.7	3.4	350	790	10	RNA-DIRECTED RNA POLYMERASE
902	4f5x	W	9.7	3.7	290	975	9	VP2 PROTEIN
903	2yia	B	9.7	3.4	350	790	10	RNA-DIRECTED RNA POLYMERASE
904	3avv	A	9.7	5.9	270	1203	10	
905	3mmp	G	9.7	4.4	263	549	11	ELONGATION FACTOR TU 2, ELONGATION FACTOR TS
906	4a8w	C	9.7	4.1	343	643	12	RNA-DIRECTED RNA POLYMERASE
907	6abd	B	9.7	4.5	298	702	13	POLYMERASE 3
908	4ieg	B	9.7	4	323	634	10	RNA-DEPENDENT RNA POLYMERASE P2
909	6kuu	B	9.7	4.3	309	705	13	POLYMERASE 3
910	3zed	A	9.6	3.3	351	765	10	RNA-DIRECTED RNA POLYMERASE
911	2yi9	B	9.6	3.4	351	771	10	RNA-DIRECTED RNA POLYMERASE
912	2jlg	C	9.6	4.1	344	651	11	RNA-DIRECTED RNA POLYMERASE
913	2jlf	A	9.6	4.1	343	654	12	RNA-DIRECTED RNA POLYMERASE
914	2yi9	D	9.5	3.3	351	771	11	RNA-DIRECTED RNA POLYMERASE
915	2yia	G	9.5	3.4	351	762	11	RNA-DIRECTED RNA POLYMERASE
916	2yi8	B	9.5	3.4	351	771	11	RNA-DIRECTED RNA POLYMERASE
917	3zed	B	9.5	3.4	352	766	11	RNA-DIRECTED RNA POLYMERASE
918	6qpg	K	9.5	4.3	304	664	10	POLYMERASE ACIDIC PROTEIN
919	2jlg	B	9.5	4.2	346	664	12	RNA-DIRECTED RNA POLYMERASE
920	2jlf	C	9.5	4.1	344	654	12	RNA-DIRECTED RNA POLYMERASE
921	1uvk	E	9.4	4.2	346	664	12	RNA-DIRECTED RNA POLYMERASE
922	5gan	A	9.4	15.8	351	2196	8	
923	4b02	C	9.4	4	342	664	11	RNA-DIRECTED RNA POLYMERASE
924	4a8q	A	9.4	4.1	344	664	12	RNA-DIRECTED RNA POLYMERASE
925	1uvk	A	9.4	4.2	345	664	12	RNA-DIRECTED RNA POLYMERASE
926	1uvk	C	9.4	4.2	344	664	12	RNA-DIRECTED RNA POLYMERASE
927	4a8o	C	9.4	4.2	347	664	12	RNA-DIRECTED RNA POLYMERASE
928	4a8s	A	9.4	4.2	345	664	12	RNA-DIRECTED RNA POLYMERASE
929	1uvm	B	9.4	4	339	664	12	RNA-DEPENDENT RNA POLYMERASE
930	4a8w	B	9.4	4.2	346	664	12	RNA-DIRECTED RNA POLYMERASE
931	4a8o	B	9.4	4.2	344	664	12	RNA-DIRECTED RNA POLYMERASE
932	1uvi	A	9.4	4.2	346	664	12	RNA-DEPENDENT RNA POLYMERASE

933	4a8q	B	9.4	4.2	343	664	12	RNA-DIRECTED RNA POLYMERASE
934	4b02	B	9.4	4.1	340	664	11	RNA-DIRECTED RNA POLYMERASE
935	4a8k	B	9.4	4.2	347	664	12	RNA-DIRECTED RNA POLYMERASE
936	4a8f	A	9.4	4.1	344	664	12	RNA-DIRECTED RNA POLYMERASE
937	4a8y	A	9.4	4.2	345	664	12	RNA-DIRECTED RNA POLYMERASE
938	4a8m	P	9.4	4.2	349	664	12	RNA-DIRECTED RNA POLYMERASE
939	1hi0	P	9.4	4.2	345	664	12	P2 PROTEIN
940	1hi8	A	9.4	4.2	346	664	12	P2 PROTEIN
941	2jlg	A	9.4	4.2	346	664	11	RNA-DIRECTED RNA POLYMERASE
942	6ty8	A	9.4	4.8	317	1196	10	RNA-DEPENDENT RNA POLYMERASE
943	1uvi	B	9.4	4.2	345	664	12	RNA-DEPENDENT RNA POLYMERASE
944	2pus	A	9.4	3.5	352	765	11	IBDV VP1 RNA-DEPENDANT RNA POLYMERASE
945	1hi0	Q	9.4	4.2	345	664	12	P2 PROTEIN
946	1hi0	R	9.4	4.2	345	664	12	P2 PROTEIN
947	1hi1	A	9.4	4.2	347	664	12	P2 PROTEIN
948	2yia	F	9.3	3.4	351	782	11	RNA-DIRECTED RNA POLYMERASE
949	2yia	H	9.3	3.4	352	790	11	RNA-DIRECTED RNA POLYMERASE
950	4a8f	B	9.3	4	346	664	11	RNA-DIRECTED RNA POLYMERASE
951	1hi1	C	9.3	4.1	346	664	12	P2 PROTEIN
952	2jl9	B	9.3	4.1	346	656	12	RNA-DIRECTED RNA POLYMERASE
953	2yia	A	9.2	3.4	349	790	11	RNA-DIRECTED RNA POLYMERASE
954	2yib	C	9.2	3.4	351	789	10	RNA-DIRECTED RNA POLYMERASE
955	2yia	C	9.2	3.4	352	790	11	RNA-DIRECTED RNA POLYMERASE
956	3avu	A	9.2	5.6	272	1203	10	
957	6qwl	K	9.1	4.3	271	539	10	POLYMERASE ACIDIC PROTEIN
958	3jb6	A	9.1	4.7	317	1196	9	RNA-DEPENDENT RNA POLYMERASE
959	6kup	B	9	4.2	294	701	13	POLYMERASE 3
960	5mps	A	8.9	4	339	1914	7	YEAST UBC4 GENE FOR UBIQUITIN-CONJUGATING ENZYME
961	3zef	E	8.8	3.7	313	1420	6	PRE-MRNA-SPLICING FACTOR 8
962	3zef	B	8.8	3.8	316	1398	6	PRE-MRNA-SPLICING FACTOR 8
963	4i43	B	8.7	3.6	318	1407	7	A1 CISTRON-SPLICING FACTOR AAR2
964	5mqf	A	8.7	4.1	334	1965	8	PRE-MRNA-PROCESSING-SPLICING FACTOR 8
965	6qnw	K	8.7	4.3	302	673	11	POLYMERASE ACIDIC PROTEIN
966	3mmp	F	8.6	4.3	270	549	11	ELONGATION FACTOR TU 2, ELONGATION FACTOR TS
967	6me0	C	8.6	4.5	273	414	10	T.EL4H RNA
968	5mq0	A	8.6	3.9	336	1914	6	YEAST UBC4 GENE FOR UBIQUITIN-CONJUGATING ENZYME
969	6j6q	A	8.5	3.9	339	1913	7	PRE-MRNA-SPLICING FACTOR 8

970	5y88	A	8.5	4.1	336	1903	7	PRE-MRNA-SPLICING FACTOR 8
971	2r7x	A	8.4	4	326	1073	10	RNA (5'-R(*UP*GP*UP*GP*AP*CP*C)-3')
972	5wsg	A	8.4	3.9	335	1931	8	PRE-MRNA-SPLICING FACTOR 8
973	2r7s	A	8.3	3.8	315	1073	10	RNA (5'-R(*UP*GP*UP*GP*CP*C)-3')
974	1mwh	A	8.3	4.3	332	1256	9	MINOR CORE PROTEIN LAMBDA 3
975	3jcm	A	8.2	4	332	2174	7	PRE-MRNA-SPLICING FACTOR 8
976	6exn	A	8.2	3.9	338	1945	7	U2 SNRNA
977	6j6h	A	8.2	3.9	339	1913	7	PRE-MRNA-SPLICING FACTOR 8
978	5ylz	A	8.1	4	341	1931	6	PRE-MRNA-SPLICING FACTOR 8
979	3jb9	A	8	4	338	1964	6	PRE-MRNA-SPLICING FACTOR SPP42
980	6id0	A	8	3.9	341	1981	7	PRE-MRNA-PROCESSING-SPLICING FACTOR 8
981	6ff4	A	8	3.8	331	2238	7	RNA-BINDING MOTIF PROTEIN, X-LINKED 2
982	6qx8	F	7.9	4.5	321	611	9	POLYMERASE ACIDIC PROTEIN
983	6qxe	F	7.9	4.5	321	611	9	NB8205
984	5amr	A	7.8	7.1	371	1657	8	RNA POLYMERASE L
985	6qx8	B	7.8	4.4	317	611	10	POLYMERASE ACIDIC PROTEIN
986	6id1	A	7.8	3.9	341	1981	7	PRE-MRNA-PROCESSING-SPLICING FACTOR 8
987	6pns	A	7.7	4.2	332	1291	10	RNA-DIRECTED RNA POLYMERASE
988	6icz	A	7.7	4	338	2253	7	PROTEIN MAGO NASHI HOMOLOG 2
989	6qx3	B	7.7	4.4	299	615	10	RNA (5'-R(P*AP*GP*CP*AP*AP*AP*AP*GP*CP*A)-3')
990	6qxe	B	7.6	4.5	314	611	10	NB8205
991	6mec	C	7.5	4.5	268	414	9	T.EL4H RNA
992	5d98	E	7.5	4.4	298	711	13	POLYMERASE ACIDIC PROTEIN
993	1muk	A	7.4	4.4	342	1256	8	MINOR CORE PROTEIN LAMBDA 3
994	6qpf	K	7.4	4.4	302	662	10	POLYMERASE ACIDIC PROTEIN
995	5gmk	A	7.4	4.1	342	1910	8	PRE-MRNA-SPLICING FACTOR 8
996	6pzk	A	7.3	3.6	296	1361	10	RNA-DIRECTED RNA POLYMERASE L
997	3j9b	I	7.3	4.4	264	440	0	POLYMERASE
998	2r7v	A	7.3	3.9	318	1073	9	RNA (5'-R(*G*GP*CP*UP*UP*U)-3')
999	6.00E+53	A	7.3	7.1	240	596	10	TELOMERASE REVERSE TRANSCRIPTASE
1000	3j9b	B	7.3	4.5	271	440	0	POLYMERASE
1001	5d9a	E	7.3	4.4	296	711	12	POLYMERASE ACIDIC PROTEIN
1002	6rr7	B	7.2	4.1	299	744	11	POLYMERASE ACIDIC PROTEIN
1003	5zwo	A	7.2	4	337	2172	6	PRE-MRNA-SPLICING FACTOR 8
1004	3kyl	A	7.2	7	244	596	9	TELOMERASE REVERSE TRANSCRIPTASE
1005	6u5o	L	7	3.9	298	1354	11	RNA-DIRECTED RNA POLYMERASE L
1006	6fhh	B	6.9	4.1	305	750	11	POLYMERASE ACIDIC PROTEIN

1007	5o9z	A	6.9	4.1	340	2185	7	PRE-MRNA-PROCESSING-SPLICING FACTOR 8
1008	6j6n	A	6.9	3.9	336	1913	7	PRE-MRNA-SPLICING FACTOR 8
1009	3avx	A	6.8	4.6	268	1203	10	
1010	6fhi	B	6.8	4.1	302	750	11	POLYMERASE ACIDIC PROTEIN
1011	2r7r	A	6.6	3.8	320	1073	10	RNA (5'-R(*UP*GP*UP*GP*AP*CP*C)-3')
1012	6tz2	A	6.6	7.3	342	1208	8	RNA-DEPENDENT RNA POLYMERASE
1013	3du5	A	6.6	6.9	233	596	9	TELOMERASE REVERSE TRANSCRIPTASE
1014	3jb7	A	6.5	6.7	345	1198	8	CPV RNA-DEPENDENT RNA POLYMERASE
1015	5d9a	H	6.4	4.4	299	711	13	POLYMERASE ACIDIC PROTEIN
1016	6bk8	A	6.4	4	333	1960	6	U2 SNRNA
1017	5m3h	B	6.3	4.1	318	750	10	POLYMERASE ACIDIC PROTEIN
1018	4au6	C	6.2	3.8	314	1071	9	RNA-DEPENDENT RNA POLYMERASE
1019	4au6	B	6.2	3.8	314	1071	9	RNA-DEPENDENT RNA POLYMERASE
1020	5lj5	A	5.8	4	335	2168	6	U5 SNRNA (SMALL NUCLEAR RNA)
1021	2r7o	A	5.7	3.8	315	1071	9	RNA-DEPENDENT RNA POLYMERASE
1022	6tz1	A	5.6	4.3	340	1208	9	RNA-DEPENDENT RNA POLYMERASE
1023	5cqg	B	5.6	7.5	227	596	9	TELOMERASE REVERSE TRANSCRIPTASE
1024	1n35	A	5.5	4.3	332	1264	9	5'-R(P*GP*GP*GP*GP*G)-3'
1025	4au6	D	5.1	3.8	313	1071	10	RNA-DEPENDENT RNA POLYMERASE
1026	5g2x	C	5.1	5.2	230	486	8	GROUP II INTRON
1027	1qai	B	5	4.2	167	251	5	DNA (5'-D(*CP*AP*TP*GP*CP*AP*TP*G)-3')
1028	1qaj	A	4.9	4.4	167	259	6	DNA (5'-D(*CP*AP*TP*GP*CP*AP*TP*G)-3')
1029	1qai	A	4.8	4	161	251	6	DNA (5'-D(*CP*AP*TP*GP*CP*AP*TP*G)-3')
1030	1d1u	A	4.8	4	160	255	6	DNA (5'-D(*CP*TP*CP*GP*TP*G)-3')
1031	4au6	E	4.8	3.8	314	1071	9	RNA-DEPENDENT RNA POLYMERASE
1032	6v86	A	4.7	3.9	301	1890	8	RNA-DIRECTED RNA POLYMERASE L
1033	4ol8	B	4.7	5.1	178	452	9	REVERSE TRANSCRIPTASE/RIBONUCLEASE H
1034	4au6	A	4.7	3.8	315	1071	9	RNA-DEPENDENT RNA POLYMERASE
1035	6po2	A	4.6	4.2	312	1244	10	RNA-DIRECTED RNA POLYMERASE
1036	1n1h	A	4.4	4.4	342	1264	8	5'-R(*AP*UP*UP*AP*GP*C)-3'
1037	3du6	A	3.7	10.4	251	596	10	TELOMERASE REVERSE TRANSCRIPTASE
1038	5cqg	A	3.7	7.2	239	596	10	TELOMERASE REVERSE TRANSCRIPTASE
1039	3du6	B	3.7	9.4	242	596	7	TELOMERASE REVERSE TRANSCRIPTASE
1040	3du5	B	3.3	7	226	596	9	TELOMERASE REVERSE TRANSCRIPTASE
1041	1dg3	A	2.7	8.3	82	540	16	
1042	2b7c	A	2.5	6.2	76	437	3	ELONGATION FACTOR 1-ALPHA
1043	4q7j	A	2.4	12.8	81	278	12	ELONGATION FACTOR TS
1044	5gm2	B	2.4	15.2	62	283	6	O-METHYLRANSFERASE
1045	3zvr	A	2.4	3.2	50	669	8	DYNAMIN-1

1046	5wp4	A	2.4	12.9	82	487	5	PHOSPHOETHANOLAMINE N-METHYLTRANSFERASE 1
1047	1aip	D	2.4	5.9	68	195	12	ELONGATION FACTOR TU
1048	5gam	A	2.4	5.7	70	593	6	U5 SNRNA
1049	1yra	B	2.3	10.4	66	261	6	ATP(GTP)BINDING PROTEIN
1050	4q7j	E	2.3	15.6	81	280	15	ELONGATION FACTOR TS
1051	4pc6	C	2.3	14.5	70	277	9	ELONGATION FACTOR TU
1052	2dby	A	2.3	9.6	58	355	5	GTP-BINDING PROTEIN
1053	1aip	C	2.2	6.3	69	195	12	ELONGATION FACTOR TU
1054	2f8l	A	2.2	10.8	72	324	8	HYPOTHETICAL PROTEIN LMO1582
1055	2p35	A	2.2	6.2	97	246	8	TRANS-ACONITATE 2-METHYLTRANSFERASE
1056	1aip	G	2.2	6.4	68	195	12	ELONGATION FACTOR TU
1057	6q56	A	2.1	4.6	58	234	9	TRNA (ADENINE(22)-N(1))-METHYLTRANSFERASE
1058	6pt0	A	2.1	6.7	85	353	2	CANNABINOID RECEPTOR 2
1059	3kkz	A	2.1	2.6	44	257	5	UNCHARACTERIZED PROTEIN Q5LES9
1060	5z9o	A	2.1	4.5	51	373	2	CYCLOPROPANE-FATTY-ACYL-PHOSPHOLIPID SYNTHASE
1061	1aip	H	2.1	6.1	66	194	12	ELONGATION FACTOR TU
1062	3mmp	C	2.1	13.8	79	643	10	ELONGATION FACTOR TU 2, ELONGATION FACTOR TS
1063	2ztt	C	2.1	9.2	50	71	4	RNA-DIRECTED RNA POLYMERASE CATALYTIC SUBUNIT
1064	3a1g	C	2.1	4.5	47	73	4	RNA-DIRECTED RNA POLYMERASE CATALYTIC SUBUNIT
1065	3a1g	A	2.1	5.5	47	73	2	RNA-DIRECTED RNA POLYMERASE CATALYTIC SUBUNIT
1066	2ztt	A	2.1	4.5	46	73	4	RNA-DIRECTED RNA POLYMERASE CATALYTIC SUBUNIT
1067	2qm8	A	2	3.1	47	320	4	GTPASE/ATPASE
1068	1efu	D	2	13.4	74	282	14	ELONGATION FACTOR TU

Table S2. NSP-12 cavities and their information predicted by CavityPlus server.

Rank	Pred. Max pKd	Pred. Avg pKd	Drug Score	Druggability	Residues
1	6.99	6.71	2315	Druggable	ASP:161:A, ASP:164:A, PHE:165:A, VAL:166:A, GLU:167:A, GLN:408:A, THR:409:A, VAL:410:A, LYS:411:A, LYS:438:A, HIS:439:A, PHE:441:A, ASP:452:A, TYR:455:A, TYR:456:A, ARG:457:A, TYR:458:A, VAL:493:A, ILE:494:A, VAL:495:A, ASN:496:A, ASN:497:A, LEU:498:A, ASP:499:A, LYS:500:A, SER:501:A, ALA:502:A, GLY:503:A, PHE:506:A, ASN:507:A, LYS:508:A, GLY:510:A, LYS:511:A, GLN:541:A, MET:542:A, ASN:543:A, LEU:544:A, LYS:545:A, TYR:546:A, ALA:547:A, ILE:548:A, SER:549:A, ALA:550:A, LYS:551:A, ASN:552:A, ARG:553:A, ALA:554:A, ARG:555:A, THR:556:A, VAL:557:A, ALA:558:A, GLY:559:A, VAL:560:A, THR:565:A, ASN:568:A, ARG:569:A, HIS:572:A, GLN:573:A, LEU:575:A, LEU:576:A, LYS:577:A, SER:578:A, ILE:579:A, ALA:580:A, ALA:581:A, ARG:583:A, VAL:588:A, ILE:589:A, GLY:590:A, THR:591:A, SER:592:A, LYS:593:A, PHE:594:A, TRP:598:A, MET:601:A, MET:615:A, GLY:616:A, TRP:617:A, ASP:618:A, TYR:619:A, PRO:620:A, LYS:621:A, CYS:622:A, ASP:623:A, ARG:624:A, LYS:676:A, THR:680:A, SER:681:A, SER:682:A, GLY:683:A, ASP:684:A, ALA:685:A, THR:686:A, THR:687:A, ALA:688:A, TYR:689:A, ASN:691:A, SER:692:A, MET:756:A, LEU:758:A, SER:759:A, ASP:760:A, ASP:761:A, ALA:762:A, VAL:763:A, VAL:792:A, PHE:793:A, MET:794:A, SER:795:A, GLU:796:A, ALA:797:A, LYS:798:A, CYS:799:A, TRP:800:A, THR:801:A, HIS:810:A, GLU:811:A, PHE:812:A, CYS:813:A, SER:814:A, GLN:815:A, HIS:816:A, PRO:830:A, TYR:831:A, PRO:832:A, ASP:833:A, SER:835:A, ARG:836:A, ILE:837:A, GLY:839:A, ALA:840:A, GLY:841:A, PHE:843:A, VAL:844:A, ASP:845:A, ASP:846:A, ILE:847:A, VAL:848:A, THR:853:A, LEU:854:A, MET:855:A, ILE:856:A, GLU:857:A, ARG:858:A, PHE:859:A, VAL:860:A, SER:861:A, LEU:862:A, ALA:863:A, ILE:864:A, ASP:865:A, ALA:866:A, TYR:915:A, TRP:916:A
2	8.57	6.81	1466	Druggable	VAL:31:A, TYR:32:A, ARG:33:A, ALA:34:A, PHE:35:A, ASP:36:A, ILE:37:A, TYR:38:A, ASN:39:A, ASP:40:A, LYS:41:A, VAL:42:A, ALA:43:A, GLY:44:A, LYS:47:A, PHE:48:A, LEU:49:A, LYS:50:A, TYR:69:A, PHE:70:A, VAL:71:A, VAL:72:A, LYS:73:A, ARG:74:A, THR:76:A, ASN:79:A, TYR:80:A, GLN:81:A, HIS:82:A, GLU:83:A, GLU:84:A, ILE:86:A, TYR:87:A, HIS:99:A, PRO:112:A,

					HIS:113:A, ILE:114:A, SER:115:A, ARG:116:A, LEU:119:A, THR:120:A, LYS:121:A, TYR:122:A, THR:123:A, ALA:125:A, ASP:126:A, VAL:202:A, GLY:203:A, VAL:204:A, LEU:205:A, THR:206:A, LEU:207:A, ASP:208:A, ASN:209:A, ASP:211:A, LEU:212:A, TRP:216:A, TYR:217:A, ASP:218:A, PHE:219:A, GLY:220:A, ASP:221:A, PHE:222:A, ILE:223:A, VAL:233:A, SER:236:A, ASP:711:A, ASN:713:A, HIS:725:A, TYR:728:A, ARG:733:A
3	8.83	6.83	160	Less druggable	ARG:33:A, ALA:34:A, PHE:35:A, ASP:36:A, ILE:37:A, TYR:38:A, ASN:39:A, VAL:42:A, PHE:48:A, LYS:50:A, VAL:71:A, VAL:72:A, LYS:73:A, ARG:74:A, THR:76:A, PHE:77:A, SER:78:A, ASN:79:A, TYR:80:A, GLN:81:A, HIS:82:A, GLU:83:A, GLU:84:A, THR:85:A, ILE:86:A, TYR:87:A, ASN:88:A, LYS:91:A, LYS:98:A, HIS:99:A, ASP:100:A, PHE:101:A, PHE:102:A, PRO:112:A, HIS:113:A, ILE:114:A, SER:115:A, ARG:116:A, LEU:119:A, THR:120:A, LYS:121:A, THR:123:A, GLY:203:A, VAL:204:A, LEU:205:A, THR:206:A, LEU:207:A, ASP:208:A, ASN:209:A, TRP:216:A, TYR:217:A, ASP:218:A, PHE:219:A, GLY:220:A, ASP:221:A, PHE:222:A, ILE:223:A
4	9.65	6.88	2190	Druggable	PHE:165:A, VAL:166:A, GLU:167:A, ASN:168:A, PRO:169:A, ASP:170:A, ILE:171:A, LEU:172:A, ARG:173:A, VAL:174:A, ALA:176:A, ASN:177:A, PRO:243:A, LEU:245:A, THR:246:A, LEU:247:A, THR:248:A, ARG:249:A, ALA:250:A, LEU:251:A, THR:252:A, ALA:253:A, GLU:254:A, SER:255:A, HIS:256:A, LEU:261:A, PRO:264:A, TYR:265:A, ILE:266:A, LYS:267:A, TRP:268:A, ASP:269:A, LEU:270:A, ASN:314:A, VAL:315:A, LEU:316:A, PHE:317:A, SER:318:A, THR:319:A, VAL:320:A, PHE:321:A, PRO:322:A, PRO:323:A, THR:324:A, SER:325:A, PHE:326:A, ARG:349:A, GLU:350:A, LEU:387:A, LEU:388:A, LEU:389:A, ASP:390:A, LYS:391:A, ARG:392:A, THR:393:A, THR:394:A, CYS:395:A, PHE:396:A, SER:397:A, VAL:398:A, ALA:399:A, TYR:453:A, ASP:454:A, TYR:455:A, TYR:456:A, ARG:457:A, TYR:458:A, ASN:459:A, LEU:460:A, PRO:461:A, THR:462:A, MET:463:A, PRO:627:A, ASN:628:A, MET:629:A, SER:664:A, TYR:674:A, VAL:675:A, LYS:676:A, PRO:677:A, GLY:678:A, ASN:791:A
5	10.74	6.95	3183	Druggable	TRP:268:A, ASP:269:A, LEU:270:A, LEU:271:A, LYS:272:A, TYR:273:A, ASP:274:A, PHE:275:A, PRO:322:A, PRO:323:A, THR:324:A, SER:325:A, PHE:326:A, GLY:327:A, PRO:328:A, LEU:329:A, VAL:330:A, ARG:331:A, LYS:332:A, PHE:340:A, VAL:341:A, VAL:342:A, SER:343:A, THR:344:A, GLY:345:A, TYR:346:A, HIS:347:A, PHE:348:A, ARG:349:A, GLU:350:A, LEU:351:A, HIS:355:A, ASP:377:A, PRO:378:A, ALA:379:A, MET:380:A, HIS:381:A,

					ALA:382:A, ALA:383:A, SER:384:A, GLY:385:A, ASN:386:A, LEU:387:A, LEU:388:A, LEU:389:A, ASP:390:A, LYS:391:A, THR:394:A, CYS:395:A, PHE:396:A, SER:397:A, VAL:398:A, ALA:399:A, ALA:400:A, PRO:537:A, ASN:628:A, CYS:659:A, ALA:660:A, LEU:663:A, SER:664:A, GLU:665:A, MET:666:A, TYR:674:A, VAL:675:A, LYS:676:A, PRO:677:A, GLY:678:A
6	10.84	6.96	1525	Druggable	PHE:165:A, VAL:166:A, ASN:168:A, PRO:169:A, ASP:170:A, ILE:171:A, LEU:172:A, ARG:173:A, VAL:174:A, TYR:175:A, ALA:176:A, ASN:177:A, LEU:178:A, GLY:179:A, GLU:180:A, ARG:181:A, ARG:183:A, PRO:243:A, LEU:245:A, THR:246:A, LEU:247:A, THR:248:A, ARG:249:A, ALA:250:A, LEU:251:A, THR:252:A, ALA:253:A, GLU:254:A, SER:255:A, HIS:256:A, LEU:261:A, LYS:263:A, PRO:264:A, TYR:265:A, ILE:266:A, LYS:267:A, TRP:268:A, LEU:270:A, ASN:314:A, VAL:315:A, LEU:316:A, PHE:317:A, SER:318:A, THR:319:A, VAL:320:A, PHE:321:A, PRO:322:A, PRO:323:A, THR:324:A, SER:325:A, PHE:326:A, ARG:349:A, GLU:350:A, THR:393:A, THR:394:A, CYS:395:A, PHE:396:A, SER:397:A, TYR:453:A, ASP:454:A, TYR:455:A, TYR:456:A, ARG:457:A, TYR:458:A, ASN:459:A, LEU:460:A, PRO:461:A, THR:462:A, MET:463:A, PRO:627:A, ASN:628:A, MET:629:A, SER:664:A, VAL:675:A, LYS:676:A, PRO:677:A, GLY:678:A, ASN:791:A
7	11.67	6.62	-518	Undruggable	VAL:31:A, TYR:32:A, ARG:33:A, ALA:34:A, PHE:45:A, ALA:46:A, LYS:47:A, PHE:48:A, TYR:122:A, ALA:125:A, ASP:126:A, LEU:127:A, VAL:128:A, TYR:129:A, ALA:130:A, LEU:131:A, ARG:132:A, HIS:133:A, PHE:134:A, ASP:135:A, GLU:136:A, GLY:137:A, ASN:138:A, CYS:139:A, ASP:140:A, THR:141:A, LEU:142:A, LYS:143:A, GLU:144:A, ASN:705:A, ALA:706:A, LEU:708:A, SER:709:A, THR:710:A, ASP:711:A, SER:772:A, GLN:773:A, GLY:774:A, SER:778:A, LYS:780:A, ASN:781:A, PHE:782:A, LYS:783:A, SER:784:A, GLU:796:A
8	9.55	5.89	-246	Undruggable	LEU:366:A, PHE:368:A, GLU:370:A, LEU:371:A, LEU:372:A, VAL:373:A, TYR:374:A, ALA:375:A, ALA:376:A, ASP:377:A, MET:380:A, HIS:381:A, PRO:505:A, PHE:506:A, TRP:509:A, GLY:510:A, LEU:514:A, TYR:515:A, SER:518:A, ILE:562:A, CYS:563:A
9	8.92	5.68	-582	Undruggable	ASN:489:A, ALA:490:A, ASN:491:A, GLN:492:A, VAL:493:A, ILE:494:A, VAL:495:A, LEU:498:A, ALA:512:A, ARG:513:A, TYR:515:A, TYR:516:A, ASP:517:A, SER:518:A, MET:519:A, SER:520:A, TYR:521:A, GLU:522:A, GLN:524:A, MET:566:A, ARG:569:A, GLN:570:A
10	8.84	5.65	-637	Undruggable	ASP:291:A, GLN:292:A, THR:293:A, HIS:295:A, CYS:301:A, LEU:302:A, ASP:303:A, ASP:304:A, ARG:305:A, CYS:306:A,

					ILE:307:A, HIS:309:A, CYS:310:A, ARG:467:A, LEU:470:A, GLU:474:A, ARG:640:A, ASN:734:A, ARG:735:A, ASP:736:A, VAL:737:A
11	8.79	5.63	38	Less druggable	VAL:410:A, LYS:411:A, PRO:412:A, GLY:413:A, PHE:440:A, PHE:441:A, PHE:442:A, ALA:443:A, GLN:444:A, ALA:448:A, ASN:543:A, LEU:544:A, LYS:545:A, TYR:546:A, ALA:547:A, ILE:548:A, SER:549:A, ALA:550:A, LYS:551:A, ASN:552:A, ARG:553:A, ALA:554:A, ARG:555:A, THR:556:A
12	8.49	5.53	35	Less druggable	ASP:235:A, ARG:305:A, ILE:466:A, ARG:467:A, GLN:468:A, LEU:469:A, LEU:470:A, PHE:471:A, VAL:472:A, VAL:473:A, GLU:474:A, GLU:729:A, CYS:730:A, LEU:731:A, TYR:732:A, ARG:733:A, ASN:734:A, ARG:735:A, ASP:736:A, VAL:737:A, ASP:738:A, THR:739:A, PHE:741:A, VAL:742:A
13	8.48	5.53	-1114	Undruggable	LEU:302:A, ASP:303:A, ASP:304:A, ARG:305:A, CYS:306:A, GLU:474:A, ASP:477:A, LYS:478:A, PHE:480:A, ASP:481:A, CYS:482:A, TYR:483:A, ASP:484:A, GLY:485:A, GLY:486:A, LEU:636:A, VAL:637:A, LEU:638:A, ALA:639:A, ARG:640:A, LYS:641:A, HIS:642:A, THR:643:A, THR:644:A, CYS:646:A, LEU:648:A, ARG:651:A, ARG:735:A, ASP:736:A, VAL:737:A, THR:739:A, VAL:742:A
14	8.32	5.47	-598	Undruggable	ASN:414:A, PHE:415:A, ASN:416:A, LYS:417:A, ASP:418:A, PHE:419:A, ASP:421:A, PHE:422:A, ALA:423:A, SER:425:A, VAL:848:A, LYS:849:A, THR:850:A, ASP:851:A, GLY:852:A, THR:853:A, MET:855:A, ILE:856:A, PHE:859:A, TYR:887:A, LYS:890:A, LEU:891:A, HIS:892:A, GLU:894:A, LEU:895:A
15	7.84	5.31	-577	Undruggable	VAL:335:A, PHE:340:A, VAL:341:A, VAL:342:A, LEU:366:A, PHE:368:A, GLU:370:A, LEU:371:A, LEU:372:A, VAL:373:A, TYR:374:A, ALA:375:A, ALA:376:A, ASP:377:A, ALA:379:A, MET:380:A, HIS:381:A, ALA:382:A, SER:384:A, GLY:385:A, LEU:401:A, PHE:504:A, PRO:505:A, PHE:506:A, LYS:508:A, TRP:509:A
16	7.76	5.28	-690	Undruggable	ASN:611:A, HIS:613:A, LEU:614:A, MET:615:A, GLY:616:A, PHE:766:A, ASN:767:A, SER:768:A, THR:769:A, TYR:770:A, ALA:771:A, SER:772:A, GLN:773:A, GLY:774:A, VAL:776:A, ALA:777:A, SER:778:A, ILE:779:A, LYS:780:A, ASN:781:A, MET:794:A, SER:795:A, GLU:796:A, ALA:797:A, LYS:798:A, CYS:799:A, TRP:800:A, THR:801:A, GLU:802:A, THR:803:A
17	7.74	5.27	-1133	Undruggable	PHE:134:A, ASP:135:A, GLU:136:A, GLY:137:A, ASN:138:A, CYS:139:A, ASP:140:A, THR:141:A, LYS:143:A, ASP:154:A, TYR:156:A, PHE:157:A, ASN:158:A, LYS:159:A, LYS:160:A, ASP:161:A, TRP:162:A, TYR:163:A, ASP:164:A, GLU:167:A, PHE:793:A
18	7.59	5.22	-607	Undruggable	PRO:328:A, ARG:331:A, ILE:333:A, VAL:335:A, VAL:342:A, SER:343:A, THR:344:A, GLY:345:A,

					ASN:356:A, GLN:357:A, ASP:358:A, VAL:359:A, ASN:360:A, LEU:361:A, HIS:362:A, SER:363:A, SER:364:A, ARG:365:A, LEU:366:A, SER:367:A, LYS:369:A, GLU:370:A, VAL:373:A, TYR:374:A, ALA:376:A, ASP:377:A, PRO:378:A, ALA:526:A, TYR:530:A, ARG:533:A, ASN:534:A, VAL:535:A, ILE:536:A, PRO:537:A, THR:538:A
19	7.06	5.04	-806	Undruggable	TYR:69:A, LEU:119:A, THR:120:A, LYS:121:A, TYR:122:A, GLU:144:A, ILE:145:A, LEU:146:A, VAL:147:A, THR:148:A, TYR:149:A, ASN:150:A, ARG:181:A, LEU:212:A, ASN:213:A
20	6.82	4.96	-812	Undruggable	LEU:251:A, ALA:253:A, GLU:254:A, SER:255:A, HIS:256:A, VAL:257:A, ASP:258:A, THR:259:A, ASP:260:A, ILE:266:A, TRP:268:A, PHE:275:A, GLU:277:A, GLU:278:A, ARG:279:A, LYS:281:A, LEU:282:A, ARG:285:A, PHE:317:A, VAL:320:A, PHE:321:A
21	6.68	4.91	-1317	Undruggable	ASN:39:A, ASP:40:A, LYS:41:A, VAL:42:A, ALA:43:A, GLY:712:A, ASN:713:A, LYS:714:A, ILE:715:A, ASP:717:A, LYS:718:A, TYR:719:A, ARG:721:A, ASN:722:A, LEU:723:A, GLN:724:A, HIS:725:A, ARG:726:A, GLU:729:A
22	6.52	4.85	-1213	Undruggable	TYR:69:A, TYR:87:A, LYS:91:A, ASP:92:A, CYS:93:A, PRO:94:A, ALA:95:A, VAL:96:A, ALA:97:A, LYS:98:A, HIS:99:A, GLN:117:A, ARG:118:A, LEU:119:A, THR:120:A, LYS:188:A, ASP:211:A, ASN:213:A, GLY:214:A, ASN:215:A, TRP:216:A
23	6.48	4.84	-1370	Undruggable	PHE:442:A, ALA:443:A, GLN:444:A, ASP:445:A, GLY:446:A, ASN:447:A, ALA:448:A, ALA:449:A, ILE:450:A, SER:451:A, ASP:452:A, TYR:453:A, TYR:455:A, LEU:544:A, LYS:551:A, ASN:552:A, ARG:553:A, ALA:554:A, ARG:555:A
24	6.39	4.81	-1227	Undruggable	PHE:415:A, TYR:420:A, VAL:424:A, PHE:428:A, PHE:429:A, LYS:430:A, GLU:431:A, GLY:432:A, SER:433:A, VAL:435:A, GLU:436:A, LEU:437:A, LYS:438:A, PHE:440:A, CYS:842:A, PHE:843:A
25	6.16	4.73	-1184	Undruggable	CYS:193:A, ASP:194:A, MET:196:A, ARG:197:A, ASN:198:A, GLY:228:A, SER:229:A, GLY:230:A, VAL:231:A, PRO:232:A, VAL:233:A, VAL:234:A, PHE:283:A, ASP:284:A, PHE:287:A, LYS:288:A, TYR:289:A, TRP:290:A, ASP:291:A, GLN:292:A, THR:293:A
26	5.57	4.53	-1302	Undruggable	TYR:122:A, CYS:139:A, ASP:140:A, THR:141:A, LEU:142:A, LYS:143:A, GLU:144:A, ILE:145:A, LEU:146:A, VAL:147:A, THR:148:A, ASP:153:A, ASP:154:A, ASP:155:A, TYR:156:A, PHE:157:A, ASN:158:A, TRP:162:A
27	5.47	4.5	-1321	Undruggable	ALA:423:A, LYS:426:A, GLY:427:A, PHE:428:A, PHE:429:A, LYS:430:A, GLU:431:A, GLY:432:A, SER:433:A, SER:434:A, VAL:435:A, LEU:838:A, GLN:875:A, GLU:876:A, TYR:877:A, ASP:879:A, VAL:880:A, LEU:883:A
